# IFN-γ-dependent regulation of intestinal epithelial homeostasis by NKT cells

**DOI:** 10.1016/j.celrep.2024.114948

**Published:** 2024-11-23

**Authors:** Marta Lebrusant-Fernandez, Tom ap Rees, Rebeca Jimeno, Nikolaos Angelis, Joseph C. Ng, Franca Fraternali, Vivian S.W. Li, Patricia Barral

**Affiliations:** 1 Centre for Inflammation Biology and Cancer Immunology, The Peter Gorer Department of Immunobiology, King’s College London, London, UK; 2 The Francis Crick Institute, London, UK; 3 Randall Centre for Cell & Molecular Biophysics, King’s College London, London, UK; 4 Institute of Structural and Molecular Biology, University College London, London, UK

**Keywords:** NKT cell, intestinal epithelial cell, intestinal organoids, IFN-γ

## Abstract

Intestinal homeostasis is maintained through the combined functions of epithelial and immune cells that collaborate to preserve the integrity of the intestinal barrier. However, the mechanisms by which immune cell populations regulate intestinal epithelial cell (IEC) homeostasis remain unclear. Here, we use a multi-omics approach to study the immune-epithelial crosstalk and identify CD1d-restricted natural killer T (NKT) cells as key regulators of IEC biology. We find that NKT cells are abundant in the proximal small intestine and show hallmarks of activation at steady state. Subsequently, NKT cells regulate the survival and the transcriptional and cellular composition landscapes of IECs in intestinal organoids, through interferon-γ (IFN-γ) and interleukin-4 secretion. *In vivo*, lack of NKT cells results in an increase in IEC turnover, while NKT cell activation leads to IFN-γ-dependent epithelial apoptosis. Our findings propose NKT cells as potent producers of cytokines that contribute to the regulation of IEC homeostasis.

## Highlights

• Intestinal NKT cells have a transcriptional program with hallmarks of basal activation
• NKT cell-derived cytokines control the viability and differentiation of IECs
• NKT cell activation *in vivo* leads to IFN-γ-dependent epithelial apoptosis

## Graphical abstract

**Figure:**
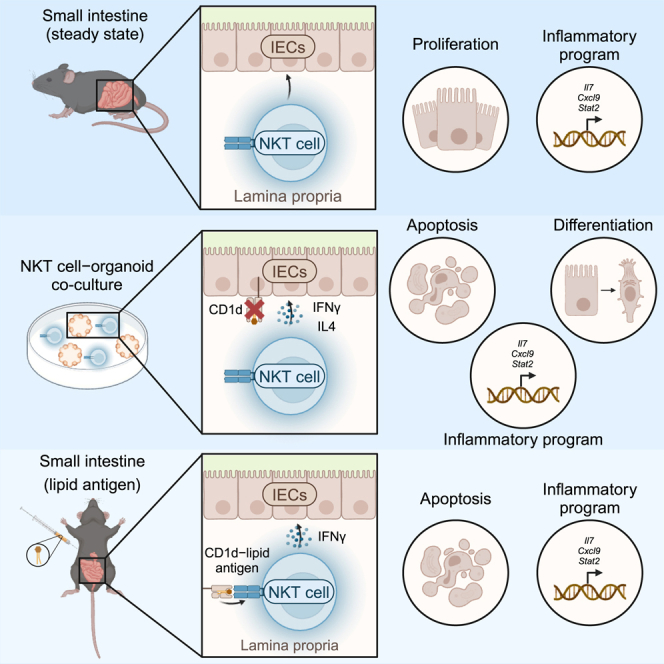

## Teaser

The crosstalk between immune and epithelial cells is vital to maintain intestinal homeostasis. Lebrusant-Fernandez et al. identify NKT cells in the small intestine as key regulators of epithelial biology by controlling the viability, transcriptional, and cellular landscapes of the intestinal epithelium.

## Introduction

The intestinal epithelium performs key functions in nutrient absorption as well as acts as a barrier to preserve the compartmentalization of the intestinal microbiota. The intestinal lining consists of a monolayer of intestinal epithelial cells (IECs) that is characterized by tight regulation and large turnover capacity. Intestinal stem cells (ISCs; located in the intestinal crypt) divide to yield new IECs and differentiate into transit-amplifying (TA) cells, which in turn adopt absorptive (enterocyte) or secretory (Paneth, goblet, tuft, enteroendocrine) cell fates. Signaling pathways such as WNT, NOTCH and the bone morphogenic protein (BMP) pathway control the balance between self-renewal and lineage commitment.^1^ The immune system plays an essential role in maintaining intestinal homeostasis, and immune cells and their secreted products can directly drive or inhibit IEC proliferation, apoptosis or differentiation.^2^ For instance, group 3 innate lymphoid cells (ILC3s) are potent producers of interleukin-22 (IL-22), which promotes IEC homeostasis and repair,^3^^,^^4^ whereas ILC1s secrete transforming growth factor-β1, which drives the expansion of CD44v6^+^ epithelial crypts.^5^ An intriguing role for immune-IEC crosstalk has been proposed for major histocompatibility complex (MHC)-dependent T cell-IEC interactions.^6^ Populations of Lgr5^+^ IECs express MHC class II and can function as antigen-presenting cells mediating T cell activation. Conversely, T cell-derived cytokines control ISC differentiation, with pro-inflammatory signals promoting and regulatory cytokines reducing differentiation.^6^ In keeping with this, elevated interferon-γ (IFN-γ) derived from intestinal immune cells drives aging-associated loss of IEC homeostasis and regeneration.^7^

The unconventional T cell family comprises several populations of tissue-resident lymphocytes that contribute to the regulation of mucosal immunity.^8^^,^^9^ Within these populations, natural killer T (NKT) cells have the unique ability to recognize through their T cell receptors (TCRs) endogenous and exogenous lipids presented by the antigen-presenting molecule CD1d.^10^ NKT cells play key roles in the regulation of tissue homeostasis and in the early initiation of immune responses. They are major producers of cytokines (e.g., IFN-γ, IL-4, IL-10) both at steady state and very early in response to an insult, regulating the recruitment, activation, and function of immune and non-immune cells.^11^^,^^12^^,^^13^ The role of NKT cells in mucosal immunity is well established.^8^ NKT cells have been proposed to regulate bacterial colonization in the gut,^14^^,^^15^^,^^16^ although their direct role in shaping the intestinal microbiota remains unclear,^17^ with contrasting results likely influenced by environmental, dietary, or experimental factors. Within the gut, NKT cells regulate serotonin release by enterochromaffin cells and subsequent gut motility,^18^ while NKT cell activation induces Paneth cell degranulation.^19^ Moreover, NKT cells can modulate the function and recruitment of other intestinal immune cells (including B cells, regulatory T cells, or ILCs), with potential consequences for intestinal homeostasis.^8^^,^^14^^,^^20^^,^^21^ NKT cells have also been proposed to regulate intestinal inflammation in humans and mice. For instance, overexpression of CD1d in transgenic mice leads to deregulated NKT cell responses and spontaneous intestinal inflammation.^22^ Also, CD1d expression is altered in the intestines of patients with inflammatory bowel disease,^23^ and in the model of oxazolone-induced colitis, NKT cells are key regulators of inflammation.^24^^,^^25^^,^^26^ Despite these data, the functional characteristics of intestinal NKT cells and if and how they regulate IEC homeostasis and differentiation remain poorly understood.

In this study, we have investigated the immune-epithelial crosstalk to define the function of NKT cells in the regulation of IEC fate and function and the mechanisms underpinning these processes. We found that NKT cells are abundant in the proximal small intestine, and they have a distinct transcriptional program showing the hallmarks of activation at steady state. To investigate the direct effects of NKT cells on IEC biology, we developed a co-culture system with intestinal NKT cells and small intestinal organoids (SIOs) and demonstrate that NKT cells are able to directly modulate the differentiation, inflammatory program, and survival/apoptosis of IECs and induce a shift in the morphology and transcriptome of intestinal organoids to acquire properties of the fetal epithelium. This NKT cell-dependent IEC regulation is mediated by cytokine secretion and independent of CD1d expression in IECs. *In vivo*, depletion of NKT cells increases the turnover of crypt epithelial cells and results in an altered inflammatory profile in IECs. Conversely, the activation of NKT cells *in vivo* leads to IFN-γ-dependent epithelial apoptosis. Our findings reveal an unrecognized role for NKT cells in regulating the transcriptional and cellular landscapes of the intestinal epithelium and bring to light a functional axis for the regulation of epithelial homeostasis.

## Results

### Intestinal NKT cells have a unique gene expression program with hallmarks of basal activation

**Figure 1:**
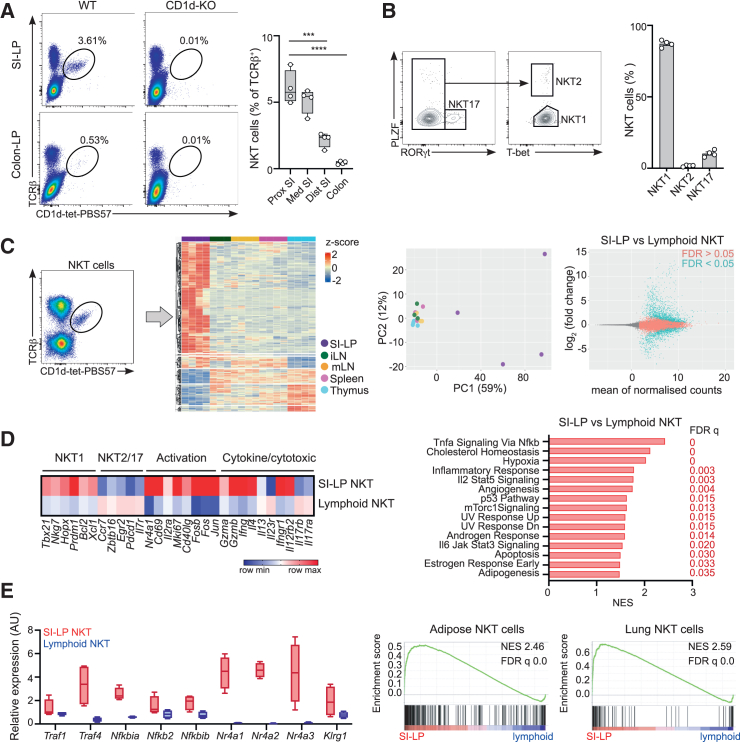
Distinct transcriptional program for intestinal NKT cells (A) Flow cytometry plots (left) and quantification (as frequency of TCR-β^+^ cells, right) for NKT cells in the small intestinal and colonic lamina propria (LP) of WT and CD1d-KO mice as indicated. NKT cell frequency is shown across the three sections of the small intestine (SI; proximal, medial, distal) as well as in the colon. Each dot is a mouse, and data are pooled from two independent experiments. Boxes show 25th to 75th percentiles, with whiskers being maximum/minimum values. ^∗∗∗^*p* < 0.001; ^∗∗∗∗^*p* < 0.0001; ANOVA with Tukey’s multiple comparisons. (B) Analysis of NKT cell subsets in the SI-LP of WT mice, showing gating strategy (left) and frequency (right) of NKT1 (T-bet^+^RORγt^−^PLZF^lo^), NKT2 (RORγt^−^PLZF^hi^T-bet^−^) and NKT17 (RORγt^+^) cells. Each dot is a mouse, and data are pooled from two independent experiments. Bars represent mean ± SEM. (C–E) Bulk RNA-seq for NKT cells purified from SI-LP, spleen, thymus, inguinal lymph node (iLN) and mesenteric lymph node (mLN). (C) Gating strategy (left). Heatmap of top differentially expressed protein-coding genes by NKT cells in the depicted tissues (center, left). Principal-component analysis (PCA) of the transcriptome of NKT cells in the depicted tissues (center, right). MA plot depicting log_2_ fold change against the mean of normalized expression counts for SI-LP vs. lymphoid NKT cells (right). Genes with FDR <0.05 are colored in blue. (D) (Left) Heatmap for fold change of selected transcripts for SI-LP vs. lymphoid NKT cells. (Right) Results of GSEA Hallmark showing top enriched gene sets. Normalized enrichment score (NES) values indicate enrichment (red bars, positive NES) in SI-LP vs. lymphoid NKT cells. (E) (Left) Relative gene expression of selected transcripts in NKT cells from SI-LP (red) vs. lymphoid tissues (blue). Boxes show 25th to 75th percentiles, with whiskers being maximum/minimum values. (Right) Enrichment plot for transcriptional signature of SI-LP NKT cells compared to signatures from NKT cells from adipose tissue^28^ and lung.^29^

To investigate the functions of NKT cells in the regulation of intestinal homeostasis, we first characterized the NKT cell population in the intestinal compartment. We identified NKT cells from the intestinal lamina propria (LP) by PBS57-loaded CD1d tetramer (CD1d-tet-PBS57; PBS57 being an analog of the prototypical lipid antigen α-galactosylceramide, αGalCer) and TCR-β co-staining (Figures 1A and S1A). NKT cells are abundant in the proximal small intestine (duodenum), and their frequency steadily decreases along the bowel (Figure 1A). As expected, these cells are absent in the tissues of CD1d-knockout (KO) mice, and as described in tissues of mice in C57BL/6 background, the majority of cells (∼90%) express the transcription factor T-bet, classifying them as NKT1 cells^11^^,^^27^ (Figures 1A and 1B). In line with this, sort-purified NKT cells from the small intestinal LP (SI-LP) secrete T helper 1 cell (Th1)-like cytokines and upregulate activation markers in response to *in vitro* stimulation with αGalCer (Figure S1B).

NKT cells exist as a predominantly tissue-resident population, and their phenotype and functions can be shaped by their local tissue environment.^30^^,^^31^^,^^32^^,^^33^ To obtain an unbiased overview of the characteristics of intestinal NKT cells, we studied the gene expression profile (by bulk RNA sequencing [RNA-seq]) of NKT cells isolated from the SI-LP and compared it with that of NKT cells isolated from lymphoid tissues from the same animals (thymus, spleen, mesenteric lymph node [mLN] and inguinal lymph node [iLN])^33^ (Figures 1C–1E). Principal-component analyses (PCAs) of the RNA-seq datasets reveal that samples from intestinal NKT cells separate from lymphoid NKT cells (Figure 1C), suggesting a distinct transcriptional program. Indeed, while we detected relatively small numbers (∼200–300) of differentially expressed genes (DEGs) between NKT cells from lymphoid tissues,^33^ SI-LP NKT cells showed more than 2,000 DEGs when compared with lymphoid NKT cells (adjusted *p* < 0.05). Using gene expression analysis, we found that intestinal NKT cells displayed enrichment of NKT1 cell markers such as *Tbx21*, *Nkg7*, or *Xcl1*, but showed lower expression of NKT2 and NKT17 markers (*Zbtb16*, *Ccr7*, *Il7r*)^29^^,^^34^ (Figure 1D), in agreement with the higher abundance of NKT2/NKT17 in lymph nodes.^33^^,^^35^ Moreover, we identified upregulated expression of genes related to cellular activation (*Nr4a1*, *Cd69*, *Il2ra*, *Fos*, *Jun*, *Cd40lg*) and secretion of cytokines (*Ifng*, *Il4*) in intestinal vs. lymphoid NKT cells (Figure 1D). In keeping with this, gene set enrichment analyses (GSEA) for MSigDB Hallmark datasets showed enrichment in intestinal NKT cells of pathways related to activation/inflammatory signaling, including tumor necrosis factor α (TNF-α) signaling via nuclear factor κB (NF-κB), inflammatory response, IL-2/STAT5 signaling or IL6-Jak-Stat3 signaling (Figure 1D). The TNF receptor superfamily-NF-κB (TNFRSF-NF-κB) axis has been described as a key marker of identity for non-lymphoid tissue T cell populations, including memory and regulatory T cells.^36^ Accordingly, T cells from barrier tissues (gut, skin) are characterized by the expression of several elements of this pathway, including transducers (*Traf1*, *Traf4*), effectors (*Nfkb1*, *Nfkb2*), and inhibitors (*Nfkbia*, *Nfkbiz*, *Nfkbid*), all of which are also found to be upregulated in intestinal NKT cells, suggesting the TNFRSF-NF-κB pathway as key to their barrier tissue identity (Figure 1E). Moreover, the degree of activation of NKT cells at steady state seems to be uniquely associated with their tissue of residence. For instance, adipose tissue NKT cells have been suggested to experience chronic endogenous activation by local tissue-derived signals,^28^^,^^37^ while lung NKT cells have a distinct transcriptional program characterized by the expression of a lung activation signature at steady state.^29^ Likewise, SI-LP NKT cells show enrichment of adipose and lung signatures with the conserved expression of genes related to activation (e.g., *Nr4a* genes) and antigen experience (e.g., *Klrg1*) (Figure 1E). Thus, together, these data indicate that intestinal NKT cells have a distinct transcriptional program and show signs of activation at baseline.

### Ablation of NKT cells/CD1d *in vivo* results in an altered proliferation and transcriptional program in IECs

**Figure 2:**
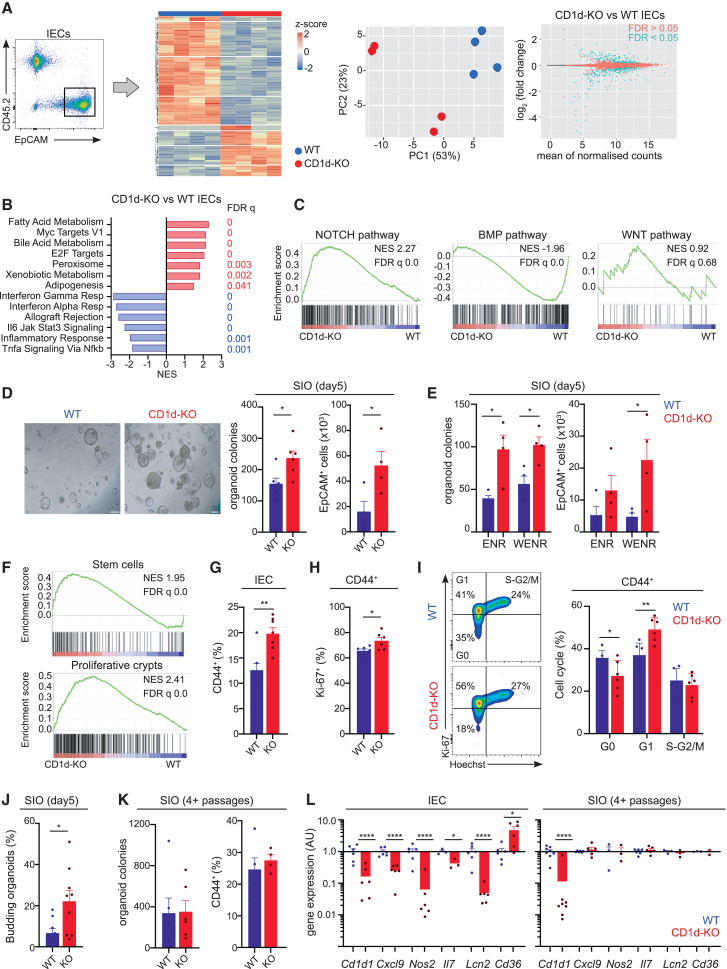
CD1d/NKT cells control the IEC transcriptome *in vivo* (A–C) Bulk RNA-seq for IECs isolated from the intestine from WT and CD1d-KO mice. (A) Gating strategy (left). Heatmap of top differentially expressed protein-coding genes by IECs from WT (blue) or CD1d-KO (red) mice (middle, left). PCA of the transcriptome of IECs (center, right). MA plot (right) depicting log_2_ fold change against mean of normalized expression counts for IECs isolated from CD1d-KO vs. WT intestines. Genes with FDR <0.05 are colored in blue. (B) Results of GSEA Hallmark showing top enriched gene sets. NES values indicate enrichment (red bars, positive NES) in CD1d-KO or WT IECs (blue bars, negative NES). (C) Enrichment plot for transcriptional signature of CD1d-KO (vs. WT) IECs compared to signatures for NOTCH,^38^ BMP,^38^ and WNT^39^ signaling pathways. (D and E) SIOs were generated from WT (blue) and CD1d-KO (red) crypts and analyzed at day 5 after seeding. (D) Representative bright-field images (left) and quantification of SIO and EpCAM^+^ cells (right) are shown. (E) SIOs were seeded in medium supplemented (WENR) or not (ENR) with WNT3a. Each dot represents counts of SIO/EpCAM^+^ cells generated from one mouse, and data are pooled from two to three independent experiments. Bars represent mean ± SEM. ^∗^*p* < 0.05, unpaired t test. (F) Enrichment plot for transcriptional signature of CD1d-KO (vs. WT) IECs compared to signatures from stem cells^40^ (top) and intestinal proliferative crypts^41^ (bottom). (G) Frequency of CD44^+^CD24^int^ cells in the SI from WT and CD1d-KO mice. Each dot is a mouse, and data are pooled from five independent experiments. Bars represent mean ± SEM. ^∗∗^*p* < 0.01, unpaired t test. (H and I) Cell-cycle analyses of CD44^+^CD24^int^ IECs stained with Ki-67 and Hoechst. (H) Frequency of Ki-67^hi^ cells. (I) Representative flow cytometry plots (left) and quantification of cells in G0 (Ki-67^−^Hoechst^−^), G1 (Ki-67^+^Hoechst^−^), or S-G2/M (Ki-67^+^Hoechst^+^). Each dot is a mouse, and data are pooled from three independent experiments. Bars represent mean ± SEM. ^∗^*p* < 0.05; ^∗∗^*p* < 0.01; unpaired t test (H) or ANOVA with Sidak’s multiple comparisons (I). (J) Frequency of budding SIOs generated from WT (blue) and CD1d-KO (red) crypts and analyzed at day 5 after seeding. Bars represent mean ± SEM. ^∗^*p* < 0.05, unpaired t test. (K) Established organoids (four or more passages) were dissociated and seeded at 2,000 cells/well. Number of recovered SIO and EpCAM^+^ cells were analyzed at day 6. Bars represent mean ± SEM. (L) Gene expression (qPCR) in freshly isolated IEC (left) or established organoids (right) from WT (blue) or CD1d-KO (red) mice. Each dot is a mouse, and data are pooled from three independent experiments. Bars represent means. ^∗^*p* < 0.05; ^∗∗∗∗^*p* < 0.0001; one-sample t test.

Given the abundance of NKT cells in the SI-LP, we speculated that they may contribute to the regulation of IEC homeostasis (Figures 2, S2, and S3). To test this, we first characterized IECs in wild-type (WT) and CD1d-KO mice (which lack NKT cells; Figures S2A and S2B). H&E staining did not demonstrate any noticeable changes in the gross morphology of the CD1d-KO mice intestine (Figure S2B). However, quantitative analyses revealed that CD1d-KO guts exhibited an altered villus:crypt ratio, suggesting possible underlying alterations in IECs from these animals (Figure S2B). To investigate the molecular alterations caused by the lack of NKT cells, we performed bulk RNA-seq from freshly isolated epithelial cells (EpCAM^+^CD45^−^CD31^−^Ter119^−^) sorted from the proximal SI crypts of WT and CD1d-KO mice (Figure 2A). These analyses revealed 612 genes that were differentially expressed between IECs isolated from CD1d-KO vs. WT guts (252 upregulated and 360 downregulated; adjusted *p* < 0.05). GSEA for MSigDB Hallmark datasets identified an enrichment in pathways related to proliferation (MYC targets, E2F targets) and metabolism (fatty acid and bile acid metabolism) in the CD1d-KO epithelia. In contrast, pathways related to immune and inflammatory responses (IFN-γ response, IFN-α response, inflammatory response, allograft response) were negatively enriched in IEC from CD1d-KO mice (Figures 2B and S2C).

The intestinal epithelium relies on the combined action of various signaling pathways (e.g., NOTCH, WNT, BMP) and key transcription factors (e.g., Atoh1, Neurog3) to control the balance between self-renewal and differentiation. ISCs depend on active NOTCH signaling to maintain their identity and proliferative capacity, while BMPs negatively regulate stemness.^1^ GSEA analyses comparing our transcriptomic dataset for WT/CD1d-KO IECs with gene sets for these pathways^38^ revealed a positive enrichment in NOTCH and a negative enrichment in BMP pathways in CD1d-KO IECs (Figure 2C). However, the expression of key regulators of epithelial identity (e.g., *Atoh1*, *Neurog3*) remained unchanged (Figure S2D). To functionally assess the impact of the altered IEC transcriptome, we harnessed the capacity of epithelial crypts to form organoids as an *in vitro* assay of intestinal stemness, and generated organoids from SI crypts from WT and CD1d-KO mice (Figure 2D). We consistently recovered more SIOs and more EpCAM^+^ cells at day 5 after seeding intestinal crypts from CD1d-KO mice in comparison to WT (Figure 2D), suggesting an increase in stem cell numbers and/or proliferative capacity of crypt cells. This phenotype was independent of media supplementation with exogenous WNT3a, as it was preserved when organoids were seeded in the presence and/or absence of exogenous WNT3a (Figure 2E), and GSEA indicated no alterations in WNT signaling^39^ in CD1d-KO IECs (Figure 2C). Comparison of our RNA-seq data with publicly available datasets^41^^,^^40^ showed an enrichment in stem cell and TA signatures as well as genes associated with intestinal crypt proliferative cells in the CD1d-KO epithelium (Figures 2F and S2E). Indeed, we detected an increased frequency of CD44^hi^CD24^int^ cells (enriched in proliferative crypts^5^^,^^42^^,^^43^) in the intestinal epithelium of CD1d-KO mice (Figures 2G and S2F). In keeping with this, cell-cycle analyses confirmed increased frequencies of Ki-67^+^ and decreased non-proliferative (G0) crypt cells (CD44^hi^CD24^int^) in the CD1d-KO epithelium (Figures 2H and 2I), suggesting that ablation of CD1d/NKT cells *in vivo* results in increased turnover of the crypt epithelium. Also, SIOs derived from CD1d-KO crypts showed significantly increased budding at 5 days after seeding vs. WT controls (Figure 2J). Through comparison of our sequencing data to published datasets generated from fetal spheroids or mature adult organoids,^44^ we found that the gene signature in CD1d-KO IECs bore more similarity to a mature adult organoid, while the fetal spheroid signature was downregulated (Figure S2G). Finally, histological analyses indicate that the numbers of OLFM4^+^ ISCs are comparable in WT and CD1d-KO guts (Figure S2H). These experiments suggest that CD1d/NKT cells control the turnover of crypt epithelial cells, yet do not alter ISC numbers.

We further investigated alterations in the functional differentiation of IECs in the CD1d-KO intestine by comparing our RNA-seq dataset to a published dataset generated from single-cell transcriptomic analyses of epithelial cells across the small intestine^40^ (Figures S3A and S3B). Enterocyte, enteroendocrine, and Paneth cell signatures were significantly enriched in CD1d-KO intestines, while tuft and goblet cell signatures were negatively enriched (Figure S3A). Despite these changes, histological analyses showed no differences in the numbers of tuft (DCLK1^+^), goblet (MUC2^+^), or enteroendocrine (CHGA^+^) cells between the WT and CD1d-KO intestines (Figure S3B). These data suggest that while NKT cells/CD1d deficiency does not lead to quantitative alterations in the numbers of secretory cells *in vivo,* it may induce changes in their qualitative transcriptional status.

The intestinal epithelium responds to signals coming from the intestinal niche, including those derived from LP immune cells.^6^^,^^7^ To test whether niche-derived signals or CD1d^45^^,^^46^ per se drive the above-mentioned changes in CD1d-KO IECs, we cultured freshly isolated intestinal crypts from WT and CD1d-KO mice for 4 or more passages and characterized the properties of established organoids. Crucially, the main phenotypes observed in the CD1d-KO gut *in vivo* are rescued when SIOs are cultured *in vitro*. Accordingly, the increased SIO formation efficiency for CD1d-KO crypts was lost when we performed colony-formation assays of established organoids (Figure 2K). Likewise, the frequency of CD44^+^ IECs was comparable for WT and CD1d-KO established SIOs, as was the frequency of proliferative or apoptotic cells (Figures 2K and S3C). Also, while the expression of immune and metabolic genes was altered in freshly isolated IECs from CD1d-KO vs. WT mice, these differences were lost in fully established organoids after several passages (Figure 2L). These data suggest that signals derived from the intestinal niche in the CD1d-KO gut (rather than intrinsic CD1d^45^^,^^46^) regulate epithelial homeostasis.

### NKT cells directly regulate the viability, differentiation, and transcriptional profile of SIOs

**Figure 3:**
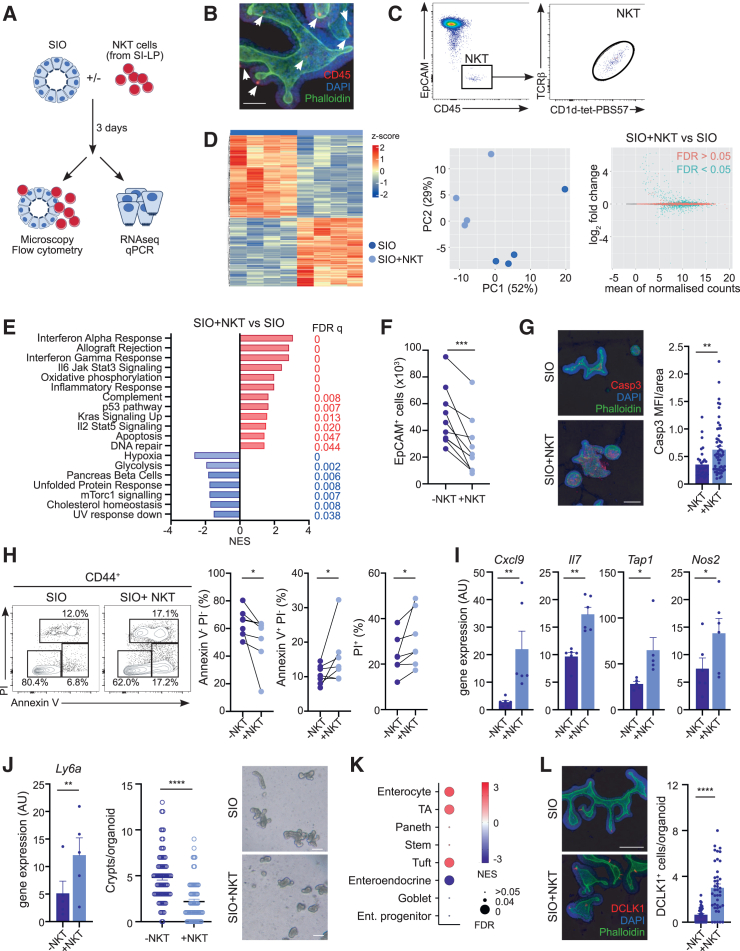
NKT cells impact SIO gene expression and survival SIO were cultured with (+NKT) or without (−NKT) intestinal NKT cells for 3 days before analyses. (A) Experimental setup. (B) Confocal microscopy images of SIO-NKT cell co-cultures showing CD45 (red), DAPI (blue), and phalloidin (green). Arrows show NKT cells. Scale bar, 50 μm. (C) Flow cytometry plots for SIO-NKT cells cultures showing CD1d-tetramer staining for NKT cells. (D) Bulk RNA-seq for SIO cultures with (SIO+NKT) or without (SIO) NKT cells. (Left) Heatmap of top differentially expressed protein-coding genes by SIO or SIO+NKT cells. (Center) PCA of the whole transcriptome of SIO±NKT cells. (Right) MA plot depicting log_2_ fold change against mean of normalized expression counts for SIO+NKT cells vs. SIO. Genes with FDR <0.05 are colored in blue. (E) Results of GSEA Hallmark showing top enriched gene sets. NES values indicate enrichment (red bars, positive NES) in SIO+NKT cells or SIO (blue bars, negative NES). (F) Number of EpCAM^+^ cells in SIO after 3-day culture alone (−NKT) or in the presence of NKT cells (+NKT). (G) Confocal microscopy images (left) and quantification of fluorescence intensity (right) for active caspase-3 staining (red) in SIO vs. SIO+NKT cell cultures. Scale bar, 50 μm. Bars represent means. (H) Representative flow cytometry staining (left) and quantification (right) of annexin V/PI populations within CD44^+^CD24^int^ IECs from SIO cultured alone (−NKT) or in the presence of NKT cells (+NKT). (I) Gene expression (qPCR) for the indicated genes in SIO cultured alone (−NKT) or in the presence of NKT cells (+NKT). Bars represent mean ± SEM. (J) *Ly6a* expression (left), crypts per organoid (center), and bright-field images (right) in SIO cultured alone (−NKT) or in the presence of NKT cells (+NKT). Bars represent mean ± SEM. (K) Enrichment plot for transcriptional signature of SIO+NKT (vs. SIO) compared to signatures from various intestinal cell types.^41^^,^^40^ (L) Confocal microscopy images (left) and quantification (right), showing DCLK1 (red), DAPI (blue), and phalloidin (green) in SIO cultured alone (−NKT) or in the presence of NKT cells (+NKT). Scale bar, 50 μm. Bars represent means. ^∗^*p* < 0.05; ^∗∗^p.0.01; ^∗∗∗^*p* < 0.001; ^∗∗∗∗^*p* < 0.0001. Statistical significance was determined by paired (F, I, J left), unpaired (G, J right, L) t test or Wilcoxon test (H). Organoids used for experiments were generated from three to four mice and data collected in at least two independent experiments.

*In vivo* IEC differentiation and function are controlled by signals derived from different cell types in the gut niche,^6^ and thus the combination of various signaling pathways can conceal and confound the identification of direct NKT cell effects. Indeed, phenotyping of myeloid and lymphoid populations in the intestine of CD1d-KO mice revealed skewed lymphoid populations in the LP (NK cells) and intraepithelial lymphocytes (TCR-β^+^ cells; Figure S4A), which could further contribute to (and/or mask) the altered IEC homeostasis in the CD1d-KO intestine. To investigate the direct contribution of NKT cells to the modulation of IEC biology, we optimized co-cultures of SIOs with primary NKT cells (isolated from the SIs of WT mice; Figures 3A–3C and S4B), which enables us to study the crosstalk between epithelial and immune cells under reductionist and controlled conditions. Cells are cultured in the presence of IL-7 and IL-15 (which support NKT cell survival and homeostasis^47^^,^^48^), but in the absence of any antigens or stimulants. In these co-cultures, established SIOs (four or more passages) are mechanically disrupted and seeded in the presence and/or absence of intestinal NKT cells. After 3 days of co-culture, we found that NKT cells surround the exterior of the organoids, and some cells appear in direct contact with IECs (Figures 3B and 3C). To investigate the effect of NKT cell-IEC crosstalk, we purified IECs for bulk RNA-seq after 3 days of co-culture (Figure 3D). NKT cells induced significant changes in the epithelial transcriptome, and PCA revealed that samples separate by culture conditions (±NKT cells). In agreement with this, 1,426 DEGs were detected between SIO and SIO+NKT cells (Figure 3D; 630 upregulated and 796 downregulated; adjusted *p* < 0.05). GSEA with MSigDB Hallmark gene sets performed on samples from SIO+NKT cells vs. SIOs (Figure 3E) identified a positive enrichment of immune-related pathways, including IFN-α and IFN-γ response, inflammatory response, or IL-2/STAT5 signaling, as well as an upregulation of apoptosis. However, NKT cell co-culture induced a downregulation mammalian target of rapamycin complex 1 signaling, which is associated with stemness and cellular proliferation (Figure 3E), and a positive enrichment in a fetal-organoid signature^44^ (Figures S4C and S4D).

To investigate the functional relevance of the transcriptional program induced by NKT cells, we measured IEC numbers and phenotypes after 3 days of SIO-NKT cell co-culture (Figures 3F–3J). We recovered fewer IECs and detected an increase in cleaved caspase 3 in SIOs after NKT cell co-culture (Figures 3F and 3G), consistent with an increase in apoptosis as detected at the transcriptional level. Moreover, using annexin V^+^ staining, we measured an increase in apoptotic cells and a decrease in viable (annexin V^−^ propidium iodide-negative [PI^−^]) cells within CD44^hi^ IECs, indicating that NKT cells induce apoptosis of crypt cells (Figure 3H). Also, in keeping with the transcriptional results, inflammation-related genes were upregulated in SIOs after NKT cell co-culture (Figure 3I). Finally, in agreement with the enriched fetal-like signature driven by NKT cells, we detected an increase in *Ly6a* expression as well as a decrease in budding in SIOs recovered after co-culture (Figure 3J). Thus, these data suggest that NKT cells alter the viability of intestinal organoids and induce a shift in their morphology and transcriptome.

Next, we investigated whether NKT cells can directly control the functional differentiation of IECs (Figures 3K, 3L, S4E, and S4F). To do this, we compared our transcriptomic datasets for SIO ± NKT cells with a published dataset generated from single-cell transcriptomic analyses of epithelial cells across the small intestine^40^ (Figure 3K). Tuft, enteroendocrine, enterocyte, and TA cell signatures were significantly altered in SIOs after NKT cell co-culture (Figures 3K and S4F). Accordingly, qPCR and immunofluorescence staining detected increased expression of the Tuft cell marker DCLK1 both at the transcriptional level and by staining of SIOs after NKT cell co-culture (Figures 3L and S4E). Also, enterocyte signatures and the enterocyte marker *Apoa4* were increased after NKT cell co-culture (Figures 3K and S4E). Altogether, these data suggest that NKT cells can directly control the differentiation, survival, and immune program of IECs.

### NKT cell-derived cytokines regulate IECs

**Figure 4:**
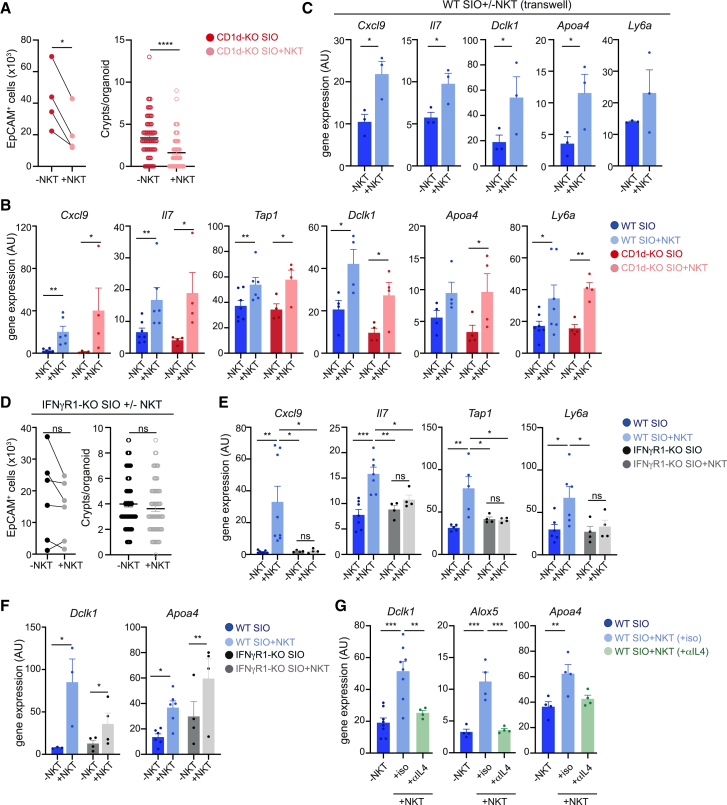
NKT cells regulate gene expression and survival of SIO through cytokine secretion (A and B) SIO derived from WT (blue) or CD1d-KO (red) mice were cultured in the presence (+NKT) or absence (−NKT) of SI-LP NKT cells for 3 days. (A) EpCAM^+^ cells (left) and crypts per organoid (right) are shown. (B) Expression of the indicated genes was measured by qPCR. (C) WT SIO were cultured with NKT cells separated in a transwell, and gene expression in SIO was measured 3 days later. (D–F) SIO derived from IFNγR1-KO (black) or WT (blue) mice were cultured in the presence (+NKT) or absence (−NKT) of NKT cells. (D) EpCAM^+^ cells (left) and crypts per organoid (right) are shown. (E and F) Expression of the indicated genes was measured by qPCR. (G) SIOs derived from WT mice were cultured in the presence (+NKT) or absence (−NKT) of NKT cells and αIL-4 (or isotype control) as indicated. Gene expression was measured by qPCR. ^∗^*p* < 0.05; ^∗∗^*p* < 0.01; ^∗∗∗^*p* < 0.001. Statistical significance was determined by paired (A left, B, C, D left, F), unpaired (A right, D right) t test or ANOVA with Tukey’s multiple comparisons (E and G). Organoids used for experiments were generated from two to four mice and data collected in at least two independent experiments. Bars represent mean ± SEM.

We next investigated the mechanism(s) by which NKT cells regulate IECs. We evaluated whether CD1d is required for the NKT cell-IEC crosstalk. We confirmed CD1d expression in IECs as well as in SIOs both at the transcriptional level (qPCR) and by flow cytometry (Figures S5A and S5B). We noted very low levels of CD1d on the surface of EpCAM^+^ cells or SIOs from WT mice, although geometric mean fluorescence intensity values were always above those detected for IECs from CD1d-KO mice, confirming that CD1d is present on the surface of IECs (Figures S5A and S5B). To functionally assess the relevance of CD1d in IEC-NKT cell crosstalk, we cultured SIOs derived from CD1d-KO mice with NKT cells and analyzed the IEC phenotype 3 days after co-culture (Figures 4A and 4B). Comparable to WT SIOs, co-culture of CD1d-KO SIOs with NKT cells led to the recovery of less budded (more spheric) organoids and reduced total numbers of IECs (Figure 4A). Moreover, immune-related genes (*Cxcl9*, *Il7*, *Tap1*), the tuft cell marker *Dclk1*, the enterocyte marker *Apoa4*, and the fetal-like marker *Ly6a* were upregulated in CD1d-KO SIOs after NKT cell co-culture (Figure 4B), suggesting a CD1d-independent IEC regulation. To test whether NKT cell-secreted factors are responsible for these phenotypes, we used a transwell essay in which sorted SI-LP NKT cells were placed in a transwell insert in contact with the organoid media to allow for the exchange of soluble factors but prevent direct NKT cell-SIO contact (Figure 4C). NKT cells located in the transwell elicited similar transcriptional changes in SIOs as to when they were in direct contact, and we observed an upregulation of immune and epithelial genes (Figure 4C).

To identify factors secreted by NKT cells that could contribute to IEC regulation, we performed predictive analyses of upstream regulators of DEGs observed in SIOs after co-culture with NKT cells as well as in IECs isolated from CD1d-KO mice. These analyses predicted IFN-γ as the top upstream regulator of upregulated DEGs in SIOs after NKT cell co-culture (positive *Z* score) and downregulated DEGs in the CD1d-KO gut (negative *Z* score; Figure S5C). Importantly, NKT cells are major producers of IFN-γ, and this cytokine has been shown to regulate IEC homeostasis.^7^^,^^49^^,^^50^ Indeed, we found that intestinal NKT cells show signs of activation and increased expression of *Ifng* (and *Il4*) when compared with lymphoid cells in homeostatic conditions (Figure 1D), and when cultured *in vitro*, we detected IFN-γ and IL-4 secretion by sort-purified SI-LP NKT cells (Figure S5D). To test whether NKT cell-derived IFN-γ controls IEC biology, we generated SIOs from IFN-γ receptor-deficient mice (IFNγR1-KO) and cultured them in the presence of NKT cells for 3 days (Figures 4D and 4E). IEC count and organoid budding remained unaffected when culturing IFNγR1-KO SIOs with NKT cells, supporting a role for NKT cell-derived IFN-γ in controlling organoid survival and morphology (Figure 4D). Moreover, induction of immune-related genes (*Cxcl9*, *Il7*, *Tap1*) and the fetal-like marker *Ly6a* were abrogated in IFNγR1-KO organoids (Figure 4E). Similar results were obtained when WT SIOs were cultured in the presence of NKT cells and an IFN-γ blocking antibody (Figure S5E). Conversely, the culture of SIOs with IFN-γ was sufficient to induce the expression of immune-related genes (*Cxcl9*, *Il7*, *Tap1*) and *Ly6a* and resulted in the recovery of fewer SIOs (Figure S5F). Lastly, we confirmed that NKT cell-derived IL-4 drives the upregulation of the tuft cell (*Dclk1* and *Alox5*) and enterocyte (*Apoa4*) hallmark genes in SIOs, as an αIL-4 antibody ablated their increase after NKT cell co-culture, while these remained unaffected in IFNγR1-KO organoids or in WT SIOs cultured in the presence of IFN-γ blocking antibody (Figures 4F, 4G, and S5E). Thus, cytokines secreted by NKT cells regulate organoid survival and morphology, as well as IEC differentiation and immune profile.

### NKT cell activation *in vivo* results in IFN-γ-dependent IEC apoptosis

**Figure 5:**
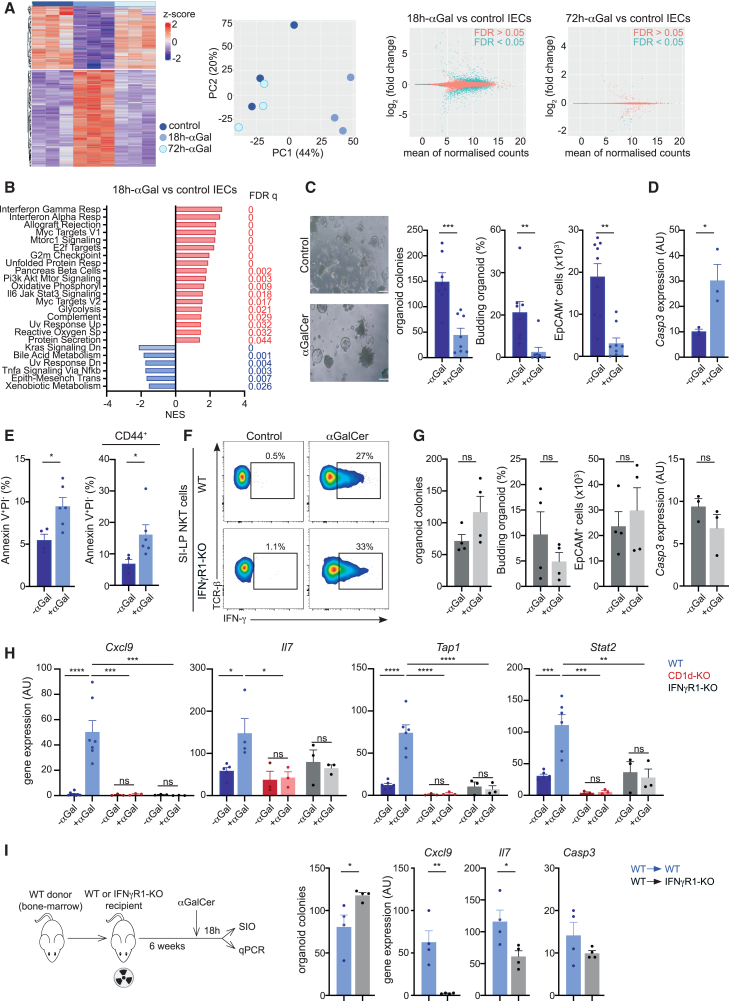
NKT cell activation *in vivo* regulates IECs (A and B) WT mice were injected with αGalCer or PBS (control) and IECs isolated for analysis at 18 h (18h-αGal) or 72 h (72h-αGal). (A) Bulk RNA-seq for IECs as indicated. (Left) Heatmap of top differentially expressed protein-coding genes. (Center) PCA of the whole transcriptome of IECs. (Right) MA plot depicting log_2_ fold change against mean of normalized expression counts for 18h-αGal vs. control IECs or 72h-αGal vs. control IECs. Genes with FDR <0.05 are colored in blue. (B) Results of GSEA Hallmark showing top enriched gene sets for 18h-αGal vs. control IECs. NES values indicate enrichment (red bars, positive NES) in 18h-αGal vs. control IECs (blue bars, negative NES). (C) WT mice were injected with αGalCer (+αGal) or PBS (−αGal) and SIO seeded 18 h later. Representative bright-field images (left) and quantification of SIO, EpCAM^+^ cells, and budding organoids (right) are shown. Bars represent means ± SEMs. Each dot are organoids generated from one mouse, and data are pooled from five independent experiments. (D and E) WT mice were injected with αGalCer (+αGal) or PBS (−αGal). (D) *Casp3* expression by qPCR and (E) quantification of apoptotic cells (annexin V^+^PI^−^) in IECs (left) and CD44^+^ cells (right) by flow cytometry are shown. Bars represent mean ± SEM. Each dot is a mouse, and data are pooled from three independent experiments. (F) Mice were injected with αGalCer or PBS (control) and SI-LP NKT cells were analyzed 5 h later. Flow cytometry plots show IFN-γ-secreting NKT cells in WT or IFNγR1-KO mice as indicated. (G) IFNγR1-KO mice were injected with αGalCer (+αGal) or PBS (−αGal) and SIO seeded 18 h later. Quantification of SIO, EpCAM^+^ cells, and budding organoids are shown. *Casp3* expression in IECs (right) was measured by qPCR. Bars represent mean ± SEM. Each dot is organoids/IECs from one mouse, and data are pooled from two independent experiments. (H) WT (blue), CD1d-KO (red), or IFNγR1-KO (gray) mice were injected with αGalCer (+αGal) or PBS (−αGal), and gene expression in IEC was measured (qPCR) 18 h later. Each dot is a mouse, and data are pooled from two to three independent experiments. (I) Experimental setup for bone marrow chimeras and quantification of SIO, as well as gene expression (qPCR) in IECs from WT→WT or WT→IFNγR1-KO chimeras 18 h after αGalCer administration. ^∗^*p* < 0.05; ^∗∗^*p* < 0.01; ^∗∗∗^*p* < 0.001; ^∗∗∗∗^*p* < 0.001. Statistical significance was determined by unpaired t test (C–G, and I) or ANOVA with Tukey’s multiple comparison test (H).

Finally, we tested the effect of *in vivo* NKT cell activation on IECs (Figure 5). We activated NKT cells by administration of the glycolipid antigen αGalCer (or PBS as control) to WT mice, and 18 or 72 h later, SI crypts were harvested and IECs were purified for RNA-seq analyses (Figures 5A, 5B, and S6A). PCA revealed that while IEC samples obtained 18 h after αGalCer administration (18h-αGal) form a distinct population (separated from the PBS control), by 72 h, αGalCer samples (72h-αGal) clustered together with the controls (Figure 5A). In line with this, we found 1,574 genes that were differentially expressed in IECs from 18h-αGal vs. control, while only 28 DEGs were detected in 72h-αGal samples, suggesting that NKT cell activation in response to αGalCer induces transient alterations in the IEC transcriptome. GSEA with MSigDB Hallmark gene sets for 18h-αGal samples identified a positive enrichment of multiple pathways including those related to response to IFN, while only one pathway (pancreas β cells) remained significantly altered (false discovery rate [FDR] <0.05) in 72h-αGal (Figures 5B and S6A). Thus, these data reveal that NKT cell activation induces transient alterations in the IEC transcriptome.

To assess the effect of NKT cell activation on IECs, we generated organoids from SI crypts from WT mice injected with αGalCer (or PBS as control; Figure 5C). We recovered fewer (budded) organoids and fewer EpCAM^+^ cells after seeding crypts from αGalCer-treated mice. In keeping with this, we measured an increase in caspase-3 expression in IECs, and annexin V staining revealed an increase in the frequency of apoptotic IECs and CD44^+^ cells after αGalCer administration (Figures 5D and 5E). Furthermore, stem cell signatures were downregulated in IECs from αGalCer-treated mice (Figure S6B). Thus, activation of intestinal NKT cells *in vivo* leads to epithelial apoptosis.

Next, we investigated the signals by which NKT cell activation controls IECs. GSEA revealed that “response to IFN-γ” was the most enriched pathway in IECs after αGalCer administration (Figure 5B), and acute exposure to IFN-γ has been shown to act on and induce apoptosis of epithelial cells in models of immune-mediated intestinal damage.^49^ Moreover, we detected secretion of IFN-γ by SI-LP NKT cells in response to αGalCer administration in WT as well as in IFNγR1-KO mice (Figures 5F, S6C, and S6D). Thus, we hypothesized that IFN-γ may underpin epithelial responses following NKT cell activation. Indeed, after the administration of αGalCer, organoid formation and budding, as well as caspase-3 expression were unaltered in IFNγR1-KO mice (Figure 5G), indicating that the IFN-γ produced in response to NKT cell activation targets the intestinal epithelium. Similarly, immune-related genes (*Cxcl9*, *Il7*, *Tap1*, *Stat2*) were strongly induced in IECs from WT mice 18 h after αGalCer administration but were abolished in IECs from αGalCer-treated CD1d-KO or IFNγR1-KO mice (Figure 5H). Finally, to confirm the IEC-specific effects driven by IFN-γ, we generated bone marrow chimeras with selective deficiency of IFNγR1 in the radioresistant compartment (WT→IFNγR1-KO). In this approach, irradiated IFNγR1-KO hosts are repopulated with WT bone marrow, such that IECs lack IFNγR1 expression while bone marrow-derived lymphocytes are of WT origin (Figure 5I). WT→WT chimeras were generated as controls. The administration of αGalCer resulted in the increased recovery of organoids in WT→IFNγR1-KO vs. control WT→WT chimeras (Figure 5I). Conversely, immune-related genes were more strongly induced in IECs from WT→WT in comparison to WT→IFNγR1-KO chimeras (Figure 5I). These data confirm that IFN-γ produced in response to NKT cell activation acts directly on the intestinal epithelium. Thus, activation of NKT cells *in vivo* results in IFN-γ-dependent targeting of the intestinal epithelium, leading to epithelial apoptosis and induction of immune-related programs in IECs.

## Discussion

To preserve barrier functions, IECs possess a great capacity for self-renewal under homeostatic conditions and of regeneration after damage. IEC developmental programs are shaped by external signals derived from epithelial and non-epithelial cells, including immune cells. Here, we identify intestinal NKT cells as key regulators of IEC biology by controlling the transcriptional and cellular landscapes of the intestinal epithelium.

NKT cells comprise a primarily tissue-resident population that is found in virtually all tissues, including in the mucosa. While previous studies have identified NKT cells within the small intestine,^14^^,^^51^ the properties and transcriptional program of these cells remained poorly characterized. Our study found that in comparison with lymphoid NKT cells, intestinal NKT cells have a distinct transcriptional program with a substantial prevalence of genes belonging to the TNFRSF-NF-κB axis. This axis has been described as a key conserved feature for barrier tissue identity in other T cell populations and proposed as a hallmark signature for tissue adaptation in non-lymphoid tissues.^36^ The enrichment of the TNFRSF-NF-κB gene signature in intestinal NKT cells suggests that these cells adapt to the intestinal environment and acquire phenotypical and functional traits distinct from cells from lymphoid tissues. In agreement with this, intestinal NKT cells show hallmarks of activation at steady state, upregulation of pathways related to inflammation, and high expression of cytokine mRNAs such as IFN-γ. Interestingly, the activation signature of intestinal NKT cells is shared by both pulmonary and adipose NKT cells.^29^^,^^37^ The signals driving this phenotype remain unclear, and while CD1d is dispensable for NKT cell survival *in vivo,*^52^ it may still be required for (or contribute to) NKT cell activation and cytokine production in the tissues in homeostatic conditions. Indeed, it is likely that cells in non-lymphoid tissues may be exposed to antigens (including commensal-derived lipids) and/or other environmental signals (e.g., cytokines, oxygen levels) that will control their activation status and ultimately dictate their functional traits at steady state and in response to insult. In support of this, intestinal microbes have been shown to regulate the numbers, phenotype, and function of NKT cells.^46^^,^^51^^,^^53^^,^^54^^,^^55^^,^^56^ Interestingly, our data also reveal an inverse correlation between NKT cell abundance (which decreases along the length of the bowel) and microbial load in the intestinal compartment (which increases along the length of the bowel). Since commensal-derived lipids negatively regulate the abundance of intestinal NKT cells,^57^ and NKT cell numbers are higher in germ-free (vs. specific pathogen-free) mice,^53^ it is tempting to speculate that commensals and their products may directly or indirectly contribute to modulating the distinct accumulation and distribution of NKT cells along the intestinal compartment.

Our data propose intestinal NKT cells as a key source of cytokines within the gut that regulate the properties of the intestinal epithelium in homeostasis. Indeed, production of cytokines by NKT cells has been shown to modulate cellular functions at different levels in homeostatic conditions. For instance, steady-state NKT cell IL-4 production regulates populations of memory CD8 T cells, immunoglobulin E production by B cells, and chemokine secretion by dendritic cells.^11^ Our data demonstrate that NKT cells are sufficient to induce apoptosis of crypt epithelial cells as well as changes in the transcriptional and cellular composition in organoids (increase in tuft cells). Conversely, the lack of NKT cells *in vivo* results in an increase in the proliferation of crypt epithelial cells and downregulation of tuft cell signatures in the CD1d-KO intestine (although the number of DCLK1^+^ cells remained unchanged). These data support a scenario in which the lack of NKT cells in the CD1d-KO intestine influences the local cytokine milieu, consequently modulating IEC survival and differentiation. It is important to note that in addition to the lack of NKT cells, we have detected alterations in other immune cell populations in the CD1d-KO intestine that may further contribute to (or mask) any direct NKT cell-mediated effects on IECs occurring *in vivo*. Our data are in line with previous reports in which Th-derived cytokines modulate IEC differentiation and the ISC pool in homeostatic conditions.^6^^,^^38^ While IFN-γ, IL-13, and IL-17 have been proposed to drive IEC differentiation (and decrease the ISC pool), IL-10 contributes to the support of the ISC niche.^6^ Also, increased production of IFN-γ by immune cells in the aged intestine has been shown to control the epithelial phenotype (increase in inflammation-related pathways, decrease in stem and TA cells) and to skew IEC differentiation.^7^

In addition to their role in the regulation of IEC homeostasis, NKT cells have been proposed to participate in intestinal inflammation and epithelial damage.^8^ Our data reveal that the activation of NKT cells by antigen administration leads to the acute release of IFN-γ that acts directly on the intestinal epithelium inducing cell death. Likewise, the release of IFN-γ by activated T cells in a model of allogeneic bone marrow transplantation leads to ISC apoptosis and intestinal damage.^49^ Also, IFN-γ contributes to reduced epithelial proliferation in murine models of colitis.^50^ Previous studies proposed key roles for NKT cells in the regulation of IECs in the context of intestinal inflammation. In the mouse model of oxazolone-induced colitis, NKT cells accumulate in the gut, while there is protection from disease in NKT cell-deficient mice or WT mice pre-treated with anti-CD1d blocking antibodies.^24^^,^^25^^,^^26^^,^^53^ In this context, CD1d expression in non-epithelial cells contributes to pathogenic NKT cell activation, while epithelial CD1d regulates the secretion of IL-10 and protection from disease.^24^^,^^26^ In our model, we propose that intestinal NKT cell activation in response to antigen may be driven by lipid presentation by myeloid cells,^14^ leading to IFN-γ secretion by NKT cells, which will target the intestinal epithelium. Moreover, while NKT cell-derived IFN-γ may be sufficient to induce an epithelial phenotype, cytokine release by NKT cells *in vivo* is known to activate other immune cells (e.g., NK cells^58^), which could in turn secrete IFN-γ and further contribute to epithelial remodeling.

Altogether, our data identify an NKT cell-epithelial axis by which NKT cells control the balance of proliferation/apoptosis and the transcriptional program of IECs. Our findings underscore the importance of the immune niche within the intestinal compartment in controlling epithelial stemness and regulating the balance between renewal and differentiation.

### Limitations of the study

Our data support an important role for NKT cell-derived cytokines in directly controlling IEC biology, but it is likely that IEC homeostasis *in vivo* is influenced by signals derived from other cell types in the gut niche, which may complement and/or compete with the signals provided by NKT cells. This may also be relevant for epithelial damage driven by NKT cell activation *in vivo*, as bystander activation of other cells in the intestinal compartment may contribute further to IFN-γ production and subsequent epithelial apoptosis. Furthermore, while CD1d is dispensable for the NKT cell-dependent regulation of IECs in our co-culture system, it is possible that CD1d-mediated IEC-NKT cell crosstalk regulates additional epithelial phenotypes (*in vitro* or *in vivo*) not investigated in our study. It is also important to note that the mice used in the experiments included in this paper are not littermates, which may have an impact in the composition of their intestinal microbiota. Changes in the microbiota are known to control NKT cell numbers and function, as well as to shape intestinal homeostasis, and may further contribute to the epithelial phenotype identified here.

## Resource availability

### Lead contact

Further information and requests for resources and reagents should be directed to and will be fulfilled by the lead contact, Patricia Barral (patricia.barral@kcl.ac.uk).

### Materials availability

This study did not generate new unique reagents.

### Data and code availability

RNA-seq data have been deposited in the NCBI’s GEO repository under the accession numbers GEO: GSE262755, GEO: GSE262532, GEO: GSE262753, and GEO: GSE262526. This paper does not report original code. Any additional information required to reanalyze the data reported in this paper is available from the lead contact upon request.

## Acknowledgments

This work was funded by the UK’s 10.13039/501100000265Medical Research Council (grant to P.B., MR/L008157/1). M.L.-F. was funded by a Francis Crick Institute-King’s College London studentship. T.a.R. was supported by a studentship from the 10.13039/501100000265Medical Research Council and King’s College London Doctoral Training Partnership in Biomedical Sciences (MR/N013700/1). R.J. was supported by a Marie Curie Intra-European Fellowship (H2020-MSCA-IF-2015-703639). The authors acknowledge technical support from the Biological Research Facility, Advanced Sequencing, Flow Cytometry, Cell Services, Histopathology, and Light Microscopy platforms of The Francis Crick Institute, which receives its core funding from 10.13039/501100000289Cancer Research UK, the 10.13039/501100000265UK Medical Research Council, and the 10.13039/100010269Wellcome Trust. We acknowledge the NIH Tetramer Core Facility for the provision of CD1d tetramers.

## Author contributions

P.B. conceptualized and supervised the study; M.L.-F. and T.a.R. conducted the experiments; R.J. generated the NKT cell RNA-seq datasets; N.A. assisted with the experimental setup and SIO generation; J.C.N. and F.F. assisted with the RNA-seq analyses; V.S.W.L. contributed to the data interpretation; P.B. wrote the paper, which was revised and edited by all authors.

## Declaration of interests

The authors declare that they have no competing financial interests.

## STAR★Methods

### Key resources table

**Table:** 

REAGENT or RESOURCE	SOURCE	IDENTIFIER
**Antibodies**		

PBS57-loaded CD1d-tetramer (mouse)	NIH tetramer Core facility	N/A
anti-mouse B220	Biolegend	Cat#: 103224; RRID: AB_313007
anti-mouse CD11b	Biolegend	Cat#: 101226; RRID: AB_830642
anti-mouse CD11c	Biolegend	Cat#: 117323; RRID: AB_830646
anti-mouse CD24	Biolegend	Cat#: 101807; RRID: AB_312840
anti-mouse CD44	Biolegend	Cat#: 103021; RRID: AB_493684
anti-mouse CD45.2	Biolegend	Cat#: 109805; RRID: AB_313442
anti-mouse CD69	Biolegend	Cat#: 104513; RRID: AB_492844
anti-mouse CD25	Biolegend	Cat#: 101910; RRID: AB_2280288
anti-mouse EpCAM (CD326)	Biolegend	Cat#: 118207; RRID: AB_1134106
anti-mouse IFNγ	Biolegend	Cat#: 505810; RRID: AB_315404
anti-mouse IL-4	Biolegend	Cat#: 504111; RRDI: AB_493321
anti-mouse Ly-6A/E (Sca-1)	Biolegend	Cat#: 122513; RRID: AB_756198
anti-mouse PLZF	Biolegend	Cat#: 145807; RRID: AB_2566166
anti-mouse RORγt	BD Biosciences	Cat#: 564722; RRID: AB_2738915
anti-mouse T-bet	Biolegend	Cat#: 644823; RRID: AB_2561760
anti-mouse TCRβ	Biolegend	Cat#: 109233; RRID: AB_2562349
anti-mouse TNFa	Biolegend	Cat#: 506327; RRID: AB_10900823
anti-mouse Ter119	Biolegend	Cat#: 116231; RRID: AB_2149212
anti-mouse CD31	Biolegend	Cat#: 102421; RRID: AB_10613457
anti-mouse NK1.1	eBioscience	Cat#: 12-5941-82; RRID: AB_466050
anti-mouse Foxp3	eBioscience	Cat#: 12-5773-82; RRID: AB_465936
anti-mouse I-A/I-E	eBioscience	Cat#: 56-5321-82; RRID: AB_494009
anti-mouse Ly6C	Biolegend	Cat#: 128041; RRDI: AB_2565852
anti-mouse Ly6G	Biolegend	Cat#: 127607; RRID: AB_1186104
anti-mouse Siglec F	BD Biosciences	Cat#: 565934; RRID: AB_2739398
anti-mouse CD64	Biolegend	Cat#: 139323; RRID: AB_2629778
anti-mouse Ki-67	Biolegend	Cat#: 652425; RRID: AB_2632693
anti-mouse TCRγδ	Biolegend	Cat#: 118107; RRID: AB_313831
Ultra-LEAF™ Purified anti-mouse IL-4	Biolegend	Cat#: 504121; RRID: AB_11149679
Ultra-LEAF™ Purified anti-mouse IFN-γ	Biolegend	Cat#: 505834; RRID: AB_11150776
Ultra-LEAF™ Purified Rat IgG1,κ Isotype Ctrl	Biolegend	Cat#: 400431; RRID: AB_11150233
anti-mouse DCAMKL1	Abcam	Cat#: ab202755; RRID: AB_10864128
anti-mouse OLFM4	Cell Signaling	Cat#: 39141T; RRID: AB_2650511
anti-mouse Chomogranin A	Abcam	Cat#: ab15160; RRID: AB_301704
Cleaved caspase-3 (Asp175)	Cell Signaling	Cat#: 9661T; RRID: AB_2341188
Goat anti-Rabbit IgG (H + L) Cross-Adsorbed	ThermoFisher	Cat#: A-21428; RRID: AB_2535849

**Chemicals, peptides, and recombinant proteins**		

Recombinant Mouse IL-15 (carrier-free)	Biolegend	Cat#: 566302
Recombinant Mouse IL-7 (carrier-free)	Biolegend	Cat#: 577802
Recombinant Mouse IFN-γ (carrier-free)	Biolegend	Cat#: 575302
anti-ARTC2 nanobody	Biolegend	Cat#: 149802
α-Galactosylceramide	Enzo Life Sciences	Cat#: ALX-306–027
Cultrex Reduced Growth Factor Basement Membrane Extract, Type 2, Pathclear	Biotechne	Cat#: 3533-010-02
B-27 Supplement	Sigma	Cat#: 17504044
EGF mouse recombinant protein	Gibco	Cat#: PMG8041
Phalloidin, iFluor 488	Abcam	Cat#: ab176753
Y-27632 dihydrochloride	Sigma	Cat#: Y0503-1MG
Brefeldin A	Biolegend	Cat#: 420601
Ionomycin	Sigma	Cat#: I0634-1MG
N-Acetyl-L-cysteine	Sigma	Cat#: A7250
Percoll	Merck	Cat#: GE17-0891-01
Phorbol 12-myristate 13-acetate (PMA)	Sigma	Cat#: P1585-1MG
ProLong™ Gold Antifade Mountant with DAPI	ThermoFisher	Cat#: P36941
DTT	Sigma	Cat#: 10197777001
Collagenase	Sigma	Cat#: C2139-100MG
DNase I	PanReac AppliChem	Cat#: A3778,0050
NADase from porcine brain	Sigma	Cat#: N9879-5G
Hoechst 33342	Biotechne	Cat#: 5117/50
Propidium iodide solution	Sigma	Cat#: P4864-10ML

**Critical commercial assays**		

123count eBeads	ThermoFisher	Cat#: 01-1234-42
Accumax	Stemcell	Cat#: 07921
Fixable viability dye	Biolegend	Cat#: 423106
Intesticult Organoid growth medium	Stemcell	Cat#: 06005
Fixation/Permeabilization solution Kit	BD Biosciences	Cat#: 554714
iScript cDNA Synthesis kit	Biorad	Cat#: 1708891
RNeasy Mini Kit	Qiagen	Cat#: 74104
iTaq Universal SyBR Green Supermix	Biorad	Cat#: 1725120
Annexin V	Biolegend	Cat#: 640924
Annexin V binding buffer	Biolegend	Cat#: 422201

**Deposited data**		

RNAseq dataset: SI NKT cells - Figure 1	This paper	GEO: GSE262755
RNAseq dataset: IEC from WT and CD1d-KO mice - Figure 2	This paper	GEO: GSE262532
RNAseq dataset: SIO^+/−^ NKT cells - Figure 3	This paper	GEO: GSE262753
RNAseq dataset: IEC from WT mice + αGalCer (18h and 72h) or control - Figure 5	This paper	GEO: GSE262526

**Experimental models: Organisms/strains**		

C57BL/6J	Wallace^59^	MGI: 3028467
CD1d1.2-KO: Del(3Cd1d2-Cd1d1)1Sbp/J	Exley et al.^60^	MGI:5582477
IFNgR1-KO: fngr1^tm1Agt^	Huang et al.^61^	MGI:63815

**Oligonucleotides**		

*Alox5* ACTACATCTACCTCAGCCTCATT GGTGACATCGTAGGAGTCCAC	Invitrogen	N/A
*Apoa4* CTAAGCAACAATGCCAAGGA GTCCTGGAAGAGGGTACTG	Invitrogen	N/A
*Cd1d* GCAGCCAGTACGCTCTTTTC ACAGCTTGTTTCTGGCAGGT	Invitrogen	N/A
*Cd36* GAACCACTGCTTTCAAAAACTGG TGCTGTTCTTTGCCACGTCA	Invitrogen	N/A
*Chga* CAAGGTGATGAAGTGCGTCC GGAGAGCCAGGTCTTGAAGT	Invitrogen	N/A
*Csf1* GCCTCCTGTTCTACAAGTGGAAG ACTGGCAGTTCCACCTGTCTGT	Invitrogen	N/A
*Cxcl9* CCTAGTGATAAGGAATGCACGATG CTAGGCAGGTTTGATCTCCGTTC	Invitrogen	N/A
*Dclk1* CGCTTCAGATCTTTCGAGGC CCGCAGACATAGCTTTCACC	Invitrogen	N/A
*Hprt* TCAGTCAACGGGGGACATAAA GGGGCTGTACTGCTTAACCAG	Invitrogen	N/A
*Il7* TCCTCCACTGATCCTTGTTC CTTCAACTTGCGAGCAGCAC	Invitrogen	N/A
*Lcn2* CCATCTATGAGCTACAAGAGAACAA CCTGTGCATATTTCCCAGAGTGA	Invitrogen	N/A
*Ly6a* CCTACCCTGATGGAGTCTGTGT CACGTTGACCTTAGTACCCAGG	Invitrogen	N/A
*Lyz1* GCCAAGGTCTACAATCGTTGTGAGTTG CAGTCAGCCAGCTTGACACCACG	Invitrogen	N/A
*Muc2* TCTACCTCACCCACAAGCTG TGGTCTGCATGCCATTGAAG	Invitrogen	N/A
*Nos2* CCACCCGAGCTCCTGGAAC CCACCCGAGCTCCTGGAAC	Invitrogen	N/A
*Tap1* GACTCCTTGCTCTCCACTCAGT AACGCTGTCACCGTTCCAGGAT	Invitrogen	N/A
*Stat2* GTTACACCAGGTCTACTCACAGA TGGTCTTCAATCCAGGTAGCC	Invitrogen	N/A
*Casp3* AAGGAGCAGCTTTGTGTGTGT AAGAGTTTCGGCTTTCCAGTC	Invitrogen	N/A

**Software and algorithms**		

FIJI (FIJI is just ImageJ)	Schindelin et al.^62^	https://imagej.net/ij/
FlowJo	TreeStar	https://www.flowjo.com/solutions/flowjo
GraphPad Prism	GraphPad Software	https://www.graphpad.com/scientific-software/prism/
GSEA	Mootha et al.^63^; Subramanian et al.^64^	https://www.gsea-msigdb.org/gsea/index.jsp
QuPath	Bankhead et al.^65^	https://qupath.github.io

### Experimental model and study participant details

Mice (WT, CD1d-KO, IFNγR1-KO) were bred under specific pathogen-free conditions at the Francis Crick Institute animal facility. Experiments were carried out using 7–10-week-old mice (males and females) with age and gender matched between genotypes. All experiments have been approved by the Francis Crick Institute and the King’s College London’s Animal Welfare and Ethical Review Body, and UK Home Office, and performed in accordance with the Animals (Scientific Procedures) Act 1986.

For *in vivo* experiments mice were injected intraperitoneally with 2 μg of αGalCer (Enzo Life Sciences) and sacrificed at the indicated time-points.

### Method details

#### Epithelial cell dissociation and crypt isolation

Crypts were isolated from the mouse duodenum following published protocols.^66^ The small intestine was harvested and rinsed in ice-cold PBS, open longitudinally, and incubated with EDTA (15mM) and DTT (1.5mM) on ice for 10 min. The intestines were then placed in EDTA (15mM) and ROCK inhibitor Y27632 (10μM) and incubated for 10 min at 37°C. The tissue was then shaken vigorously, and the supernatant (containing epithelial cells) was recovered and filtered through a 70μm filter. Samples were centrifuged at 1000rpm, 5min, 4°C to wash the cells, which results in a ‘crypt-enriched’ epithelial preparation. To generate single cell suspension, crypts were incubated in Advanced DMEM/F-12 medium containing dispase/collagenase (1 mg/ml) for 10 min at 37^°^C, with manual shaking every 2 min. Single cell suspensions were sequentially passed through 70 and 40μm filters.

#### Intestinal organoid generation and passage

Following crypt isolation from the duodenum between 200 and 500 crypts were resuspended in Cultrex Basement membrane extract (BME, Bio-Techne) and placed into a 37°C incubator with 5% CO2 for 20-30min to allow the BME to polymerise. Once the BME solidified, prewarmed organoid culture media (Intesticult, StemCell Technologies) was added to the wells. Media was topped up on day 3 by adding fresh organoid culture media and organoids were passaged every 5–7 days. The Rho kinase inhibitor Y-27632 (Sigma) was added to the culture during the first week of crypt isolation and single cell dissociation. For passaging, 1mL of cold medium was added directly on top of the BME domes which were disrupted by scraping against the plate bottom and by repeated pipetting. Organoids were further disrupted by pipetting with a glass Pasteur pipette and washed in 5mL of cold medium and spun at 1200rpm for 5 min at 4°C. The supernatant was aspirated, and organoids were resuspended in fresh BME and plated as above.

For organoid culture in ENR/WENR medium crypts were resuspended in BME as above and cultured in the following medium: the basic culture medium (ENR) contained advanced DMEM/F12 supplemented with penicillin/streptomycin, 10 mM HEPES, 2 mM Glutamax, B27 (all from Life Technologies) and 1.25 mM N-acetylcysteine (Sigma; Cat: A7250) supplemented with murine recombinant EGF (0.5 μg/mL; Gibco). R-spondin1-conditioned medium and Noggin-conditioned medium were obtained from the Cell Services platform at the Francis Crick Institute. The ‘WENR’ medium was generated by using ENR as a base medium with WNT3a-conditioned medium (obtained from Cell Services at the Francis Crick Institute).

For organoid formation assays, crypts were counted using a brightfield microscope and 200 crypts were seeded in 20μL of BME in individual wells of a 48-well plate and cultured in Intesticult/ENR/WENR medium for 5 days until counted. To perform colony formation assays in serially passaged organoids, 2 wells of stabilised organoids were harvested, resuspended in Accumax cell dissociation reagent (StemCell technologies) and incubated at room temperature for 8 min. After washing, 2000 single cells were counted and seeded in BME as described above.

#### Isolation of lamina propria lymphocytes

Intestines were dissected, cut open longitudinally and washed in ice-cold PBS to remove fecal content. Tissues were digested for 20 min at 37°C in complete IMDM medium (5% FCS) containing DTT (2mM), EDTA (5mM) and NADase (6 mg/ml). Media was discarded and intestines were washed with PBS while manually shaking for 30 s to detach any remaining epithelial cells. The small intestine was cut into 2cm pieces and digested in complete medium containing DNase I (0.1 mg/mL), collagenase VIII (1 mg/mL) and NADase (6 mg/ml). The tissues were passed through a 100μm cell strainer and washed with ice-cold IMDM. Lymphocytes were enriched by Percoll gradient centrifugation.

#### Flow cytometry and cell sorting

Flow cytometry staining was performed in FACS buffer (1% FBS, 1% BSA, 0.02% sodium azide) using the following anti-mouse antibodies from Biolegend unless specified otherwise (clones are indicated in brackets): B220 (RA3-6B2), CD11b (M1/70), CD11c (N418), TCRβ (H57-597), CD69 (H1.2F3), CD25 (PC61), IFN-γ (XMG1.2), IL-4 (11B11), TNF (MP6-XT22), PLZF (9E12), RORγt (Q31-378, BD Biosciences), T-bet (4B10), CD45.2 (104), EpCAM (9C4), CD24 (30-F1), CD44 (IM7), Ter119 (TER-119), CD31 (390), CD8 (53–6.7), NK1.1 (PK136, eBioscience), Foxp3 (FJK-16s, eBioscience), MHC-II (I-A/I-E, M5/114.15.2, eBioscience), Ly-6C (HK1.4), Ly-6G (1A8), Siglec-F (E50-2440, BD Biosciences), CD64 (X54-5/7.1), Ki-67 (16A8), TCRγδ (GL3). CD1d tetramers (loaded with PBS-57; CD1d-tet-PBS-57) were provided by the NIH Tetramer Facility. For intracellular staining, cells were fixed and permeabilised with Fixation/Permeabilization Solution Kit (BD Biosciences) according to manufacturer’s protocol. Dead cells were detected either by incubating the cell suspensions with Zombie fixable viability kit (Biolegend) for 10 min prior to surface antibody staining or by adding DAPI 10 min prior to flow cytometry analysis. For detection of apoptosis, cells were stained with Annexin V apoptosis detection kit (Biolegend) and Propidium Iodide. For cell cycle analyses cells were stained with Ki-67 and Hoechst 33342. Flow cytometry data were collected on a BD Fortessa flow cytometer and were analyzed with FlowJo software.

For sorting of intestinal NKT cells, mice were injected with anti-ARTC2 nanobody (Biolegend) 15 min before tissues were harvested. Cells were sorted as TCRβ^+^ CD1d-tet-PBS-57^+^Zombie^−^B220^−^CD11b^−^CD11c^−^CD8^−^ cells.

For epithelial cell sorting, cells were sorted as EpCAM^+^CD45^−^CD31^-^Ter119^−^Zombie^-^ cells.

#### NKT cell-SIO co-cultures

For SIO-NKT cell co-culture, NKT cells were isolated and sorted from the SI lamina propria as described above. Organoids were passaged and split as usual, and 5,000–9,000 SI-LP NKT cells were added per well. Intesticult organoid culture media was supplemented with 2-mercaptoethanol (14.3μM), IL-15 (1 ng/mL) and IL-7 (10 ng/mL).

#### NKT cell stimulation

Bone marrow-derived dendritic cell (BMDCs), were generated using standard protocols^45^ and pulsed with 100 ng/mL αGalCer for 6h. Cells were washed and cultured with NKT cells sort-purified from lamina propria at a 1:1 ratio over-night. Brefeldin A (5μg/ml, Biolegend) was added for the last 2 h of co-culture before the cells were harvested for analysis.

#### Confocal microscopy imaging of organoids

Newly passaged organoids were grown in 8-well chamber-slides (Nunc Lab-Tek) for 3–4 days before performing the staining. Media was removed and organoids were washed with PBS before fixing with 10% formalin for 20 min. The fixative was removed, and ammonium chloride (50mM) was added for 5 min for quenching. Organoids were washed with PBS, permeabilised with 0.8% Triton X-100, and blocked with PBS 1% BSA. Samples were incubated with anti-mouse primary antibodies diluted in blocking buffer overnight at 4°C: DCLK1 (EPR6085, Abcam), Cleaved caspase 3 (Asp175, Cell Signaling), CD45 (104, Biolegend). Samples were washed with PBS and incubated for 1 h with secondary antibodies and Phalloidin (Abcam). Organoids were washed and mounted with ProLong Gold antifade mountant with DAPI. Images were acquired on a Zeiss 710 upright confocal microscope. Images were processed with FIJI or QuPath.

#### qPCR

RNA extraction was performed using RNeasy Mini Kit (Qiagen). cDNA was synthesised with iScript Select cDNA Synthesis Kit (Bio-Rad) and gene expression was determined with iTaq Universal SyBR Green Supermix (Bio-Rad) and the primers included in the key resources table. Reactions were run in a real-time PCR system (ABI7900HT; Applied Biosystems).

#### Immunohistochemistry

Small intestines were cleaned from mesenteric fat, flushed with ice-cold PBS to remove feces, opened longitudinally and fixed by submerging in 10% formalin for 24 h. Formalin was then discarded, and the tissue was washed three times with PBS before submerging into 70% ethanol for 1h. Gut rolls were formed and embedded in paraffin. Tissue sections were cut into 3μm thick sections with a microtome, floated into a water bath at 40°C and placed onto microscopy slides. Slides were allowed to air dry for 1h before baking at 60°C for 1h.

For staining, paraffin-embedded tissue was deparaffinised in xylene and rehydrated in graded series of ethanol. Antigen retrieval was performed with sodium citrate buffer. Slides were blocked and incubated overnight with primary antibodies at 4C: OLFM4 (D6Y5A, Cell Signaling), CHGA (ab15160, Abcam), DCAMKL1 (ab31704, Abcam). Slides were incubated with secondary antibody for 1h followed by peroxidase substrate and counterstaining with Hematoxylin solution.

#### RNA sequencing and data analysis

Intestinal NKT cells were sorted from WT mice (in parallel to NKT cells sorted from lymphoid tissues from the same animals^33^). For IEC, 8 samples were sequenced obtained from 4 WT to 4 CD1d-KO mice. For SIO 8 samples were sequenced, 4 from WT SIO and 4 from WT SIO co-cultured for 3 days with SI-LP NKT cells. For αGalCer experiments, 3 mice/group were injected with PBS (control) or αGalCer and samples were collected at 18h or 72h for analysis. Cells were sort-purified for RNA extraction and sequencing. RNA was extracted using the Qiagen RNeasy mini kit (Qiagen) following the manufacturer’s protocol.

Libraries were generated using KAPA mRNA HyperPrep Kit (Roche – for WT/CD1d-KO IECs and SIO), Nugen Ovation ultralow kit (for LP NKT cells) or NEBNext Ultra II Directional kit (for IECs +/− αGalCer) and run on an Illumina NovaSeq 6000 system. Gene expression was quantified from raw FastQ files mapped against the release GRCm39 of the mouse genome with HISAT2. QC of alignment was performed with Picard Tools (MarkDuplicates, CollectAligmentSummaryMetrics and CollectRnaSeqMetrics tools) to generate alignment metrics that were then inspected with MultiQC. The raw count data was then imported into R and analyzed using DESeq2 to identify differentially expressed genes between genotypes or treatments. An adjusted *p* value threshold of 0.05 was applied to provide lists of differentially expressed genes (DEGs). Gene set enrichment analysis (GSEA) analysis on a pre-ranked list of genes was performed using the Broad institute GSEA tool (software.broadinstitute.org/gsea/index.jsp).

### Quantification and statistical analysis

Statistical tests were performed using Prism GraphPad software. Comparison between groups were performed using Student’s t-tests, one-way, two-way ANOVA tests as appropriate unless otherwise stated. Values of *p* < 0.05 were considered statistically significant.

## Supplemental information

Document S1. Figures S1–S6

Document S2. Article plus supplemental information

## References

[1] Beumer J., Clevers H. (2021). Cell fate specification and differentiation in the adult mammalian intestine. Nat. Rev. Mol. Cell Biol..

[2] Hageman J.H., Heinz M.C., Kretzschmar K., van der Vaart J., Clevers H., Snippert H.J. (2020). Intestinal Regeneration: Regulation by the Microenvironment. Dev. Cell.

[3] Lindemans C.A., Calafiore M., Mertelsmann A.M., O’Connor M.H., Dudakov J.A., Jenq R.R., Velardi E., Young L.F., Smith O.M., Lawrence G. (2015). Interleukin-22 promotes intestinal-stem-cell-mediated epithelial regeneration. Nature.

[4] Gronke K., Hernández P.P., Zimmermann J., Klose C.S.N., Kofoed-Branzk M., Guendel F., Witkowski M., Tizian C., Amann L., Schumacher F. (2019). Interleukin-22 protects intestinal stem cells against genotoxic stress. Nature.

[5] Jowett G.M., Norman M.D.A., Yu T.T.L., Rosell Arévalo P., Hoogland D., Lust S.T., Read E., Hamrud E., Walters N.J., Niazi U. (2021). ILC1 drive intestinal epithelial and matrix remodelling. Nat. Mater..

[6] Biton M., Haber A.L., Rogel N., Burgin G., Beyaz S., Schnell A., Ashenberg O., Su C.W., Smillie C., Shekhar K. (2018). T Helper Cell Cytokines Modulate Intestinal Stem Cell Renewal and Differentiation. Cell.

[7] Omrani O., Krepelova A., Rasa S.M.M., Sirvinskas D., Lu J., Annunziata F., Garside G., Bajwa S., Reinhardt S., Adam L. (2023). IFNgamma-Stat1 axis drives aging-associated loss of intestinal tissue homeostasis and regeneration. Nat. Commun..

[8] Brailey P.M., Lebrusant-Fernandez M., Barral P. (2020). NKT cells and the regulation of intestinal immunity: a two-way street. FEBS J..

[9] Fan X., Rudensky A.Y. (2016). Hallmarks of Tissue-Resident Lymphocytes. Cell.

[10] Mori L., Lepore M., De Libero G. (2016). The Immunology of CD1- and MR1-Restricted T Cells. Annu. Rev. Immunol..

[11] Lee Y.J., Holzapfel K.L., Zhu J., Jameson S.C., Hogquist K.A. (2013). Steady-state production of IL-4 modulates immunity in mouse strains and is determined by lineage diversity of iNKT cells. Nat. Immunol..

[12] Gaya M., Barral P., Burbage M., Aggarwal S., Montaner B., Warren Navia A., Aid M., Tsui C., Maldonado P., Nair U. (2018). Initiation of Antiviral B Cell Immunity Relies on Innate Signals from Spatially Positioned NKT Cells. Cell.

[13] Lynch L., Michelet X., Zhang S., Brennan P.J., Moseman A., Lester C., Besra G., Vomhof-Dekrey E.E., Tighe M., Koay H.F. (2015). Regulatory iNKT cells lack expression of the transcription factor PLZF and control the homeostasis of T(reg) cells and macrophages in adipose tissue. Nat. Immunol..

[14] Sáez de Guinoa J., Jimeno R., Gaya M., Kipling D., Garzón M.J., Dunn-Walters D., Ubeda C., Barral P. (2018). CD1d-mediated lipid presentation by CD11c(+) cells regulates intestinal homeostasis. EMBO J..

[15] Nieuwenhuis E.E., Matsumoto T., Lindenbergh D., Willemsen R., Kaser A., Simons-Oosterhuis Y., Brugman S., Yamaguchi K., Ishikawa H., Aiba Y. (2009). Cd1d-dependent regulation of bacterial colonization in the intestine of mice. J. Clin. Invest..

[16] Shen S., Prame Kumar K., Stanley D., Moore R.J., Van T.T.H., Wen S.W., Hickey M.J., Wong C.H.Y. (2018). Invariant Natural Killer T Cells Shape the Gut Microbiota and Regulate Neutrophil Recruitment and Function During Intestinal Inflammation. Front. Immunol..

[17] Lin Q., Kuypers M., Liu Z., Copeland J.K., Chan D., Robertson S.J., Kontogiannis J., Guttman D.S., Banks E.K., Philpott D.J., Mallevaey T. (2022). Invariant natural killer T cells minimally influence gut microbiota composition in mice. Gut Microb..

[18] Luo J., Chen Z., Castellano D., Bao B., Han W., Li J., Kim G., An D., Lu W., Wu C. (2023). Lipids regulate peripheral serotonin release via gut CD1d. Immunity.

[19] Farin H.F., Karthaus W.R., Kujala P., Rakhshandehroo M., Schwank G., Vries R.G., Kalkhoven E., Nieuwenhuis E.E., Clevers H. (2014). Paneth cell extrusion and release of antimicrobial products is directly controlled by immune cell-derived IFN-gamma. J. Exp. Med..

[20] Saez de Guinoa J., Jimeno R., Farhadi N., Jervis P.J., Cox L.R., Besra G.S., Barral P. (2017). CD1d-mediated activation of group 3 innate lymphoid cells drives IL-22 production. EMBO Rep..

[21] Clancy-Thompson E., Chen G.Z., LaMarche N.M., Ali L.R., Jeong H., Crowley S.J., Boelaars K., Brenner M.B., Lynch L., Dougan S.K. (2019). Transnuclear mice reveal Peyer's patch iNKT cells that regulate B-cell class switching to IgG1. EMBO J..

[22] Liao C., Zimmer M.I., Shanmuganad S., Yu H., Cardell S.L., Wang C. (2012). dysregulation of CD1d-restricted type ii natural killer T cells leads to spontaneous development of colitis in mice. Gastroenterol..

[23] Perera L., Shao L., Patel A., Evans K., Meresse B., Blumberg R., Geraghty D., Groh V., Spies T., Jabri B., Mayer L. (2007). Expression of nonclassical class I molecules by intestinal epithelial cells. Inflamm. Bowel Dis..

[24] Iyer S.S., Gensollen T., Gandhi A., Oh S.F., Neves J.F., Collin F., Lavin R., Serra C., Glickman J., de Silva P.S. (2018). Dietary and Microbial Oxazoles Induce Intestinal Inflammation by Modulating Aryl Hydrocarbon Receptor Responses. Cell.

[25] Heller F., Fuss I.J., Nieuwenhuis E.E., Blumberg R.S., Strober W. (2002). Oxazolone colitis, a Th2 colitis model resembling ulcerative colitis, is mediated by IL-13-producing NK-T cells. Immunity.

[26] Olszak T., Neves J.F., Dowds C.M., Baker K., Glickman J., Davidson N.O., Lin C.S., Jobin C., Brand S., Sotlar K. (2014). Protective mucosal immunity mediated by epithelial CD1d and IL-10. Nature.

[27] Engel I., Seumois G., Chavez L., Samaniego-Castruita D., White B., Chawla A., Mock D., Vijayanand P., Kronenberg M. (2016). Innate-like functions of natural killer T cell subsets result from highly divergent gene programs. Nat. Immunol..

[28] LaMarche N.M., Kane H., Kohlgruber A.C., Dong H., Lynch L., Brenner M.B. (2020). Distinct iNKT Cell Populations Use IFNgamma or ER Stress-Induced IL-10 to Control Adipose Tissue Homeostasis. Cell Metabol..

[29] Murray M.P., Engel I., Seumois G., Herrera-De la Mata S., Rosales S.L., Sethi A., Logandha Ramamoorthy Premlal A., Seo G.Y., Greenbaum J., Vijayanand P. (2021). Transcriptome and chromatin landscape of iNKT cells are shaped by subset differentiation and antigen exposure. Nat. Commun..

[30] Thomas S.Y., Scanlon S.T., Griewank K.G., Constantinides M.G., Savage A.K., Barr K.A., Meng F., Luster A.D., Bendelac A. (2011). PLZF induces an intravascular surveillance program mediated by long-lived LFA-1-ICAM-1 interactions. J. Exp. Med..

[31] Gensollen T., Lin X., Zhang T., Pyzik M., See P., Glickman J.N., Ginhoux F., Waldor M., Salmi M., Rantakari P., Blumberg R.S. (2021). Embryonic macrophages function during early life to determine invariant natural killer T cell levels at barrier surfaces. Nat. Immunol..

[32] Aguiar C.F., Corrêa-da-Silva F., Gonzatti M.B., Angelim M.K., Pretti M.A., Davanzo G.G., Castelucci B.G., Monteiro L.B., Castro G., Virgilio-da-Silva J.V. (2023). Tissue-specific metabolic profile drives iNKT cell function during obesity and liver injury. Cell Rep..

[33] Jimeno R., Lebrusant-Fernandez M., Margreitter C., Lucas B., Veerapen N., Kelly G., Besra G.S., Fraternali F., Spencer J., Anderson G., Barral P. (2019). Tissue-specific shaping of the TCR repertoire and antigen specificity of iNKT cells. Elife.

[34] Lee Y.J., Starrett G.J., Lee S.T., Yang R., Henzler C.M., Jameson S.C., Hogquist K.A. (2016). Lineage-Specific Effector Signatures of Invariant NKT Cells Are Shared amongst gammadelta T, Innate Lymphoid, and Th Cells. J. Immunol..

[35] Lee Y.J., Wang H., Starrett G., Phuong V., Jameson S., Hogquist K. (2015). Tissue-Specific Distribution of iNKT Cells Impacts Their Cytokine Response. Immunity.

[36] Miragaia R.J., Gomes T., Chomka A., Jardine L., Riedel A., Hegazy A.N., Whibley N., Tucci A., Chen X., Lindeman I. (2019). Single-Cell Transcriptomics of Regulatory T Cells Reveals Trajectories of Tissue Adaptation. Immunity.

[37] Kane H., LaMarche N.M., Ní Scannail Á., Garza A.E., Koay H.F., Azad A.I., Kunkemoeller B., Stevens B., Brenner M.B., Lynch L. (2022). Longitudinal analysis of invariant natural killer T cell activation reveals a cMAF-associated transcriptional state of NKT10 cells. Elife.

[38] Lindholm H.T., Parmar N., Drurey C., Campillo Poveda M., Vornewald P.M., Ostrop J., Díez-Sanchez A., Maizels R.M., Oudhoff M.J. (2022). BMP signaling in the intestinal epithelium drives a critical feedback loop to restrain IL-13-driven tuft cell hyperplasia. Sci. Immunol..

[39] Sansom O.J., Meniel V.S., Muncan V., Phesse T.J., Wilkins J.A., Reed K.R., Vass J.K., Athineos D., Clevers H., Clarke A.R. (2007). Myc deletion rescues Apc deficiency in the small intestine. Nature.

[40] Haber A.L., Biton M., Rogel N., Herbst R.H., Shekhar K., Smillie C., Burgin G., Delorey T.M., Howitt M.R., Katz Y. (2017). A single-cell survey of the small intestinal epithelium. Nature.

[41] Merlos-Suárez A., Barriga F.M., Jung P., Iglesias M., Céspedes M.V., Rossell D., Sevillano M., Hernando-Momblona X., da Silva-Diz V., Muñoz P. (2011). The intestinal stem cell signature identifies colorectal cancer stem cells and predicts disease relapse. Cell Stem Cell.

[42] Wang F., Scoville D., He X.C., Mahe M.M., Box A., Perry J.M., Smith N.R., Lei N.Y., Davies P.S., Fuller M.K. (2013). Isolation and characterization of intestinal stem cells based on surface marker combinations and colony-formation assay. Gastroenterol..

[43] Nefzger C.M., Jardé T., Rossello F., Horvay K., Knaupp A., Powell D., Chen J., Abud H., Polo J. (2016). A Versatile Strategy for Isolating a Highly Enriched Population of Intestinal Stem Cells. Stem Cell Rep..

[44] Mustata R.C., Vasile G., Fernandez-Vallone V., Strollo S., Lefort A., Libert F., Monteyne D., Pérez-Morga D., Vassart G., Garcia M.I. (2013). Identification of Lgr5-independent spheroid-generating progenitors of the mouse fetal intestinal epithelium. Cell Rep..

[45] Brailey P.M., Evans L., López-Rodríguez J.C., Sinadinos A., Tyrrel V., Kelly G., O’Donnell V., Ghazal P., John S., Barral P. (2022). CD1d-dependent rewiring of lipid metabolism in macrophages regulates innate immune responses. Nat. Commun..

[46] Evans L., Barral P. (2024). CD1 molecules: Beyond antigen presentation. Mol. Immunol..

[47] Park J.Y., Won H.Y., DiPalma D.T., Kim H.K., Kim T.H., Li C., Sato N., Hong C., Abraham N., Gress R.E., Park J.H. (2022). In vivo availability of the cytokine IL-7 constrains the survival and homeostasis of peripheral iNKT cells. Cell Rep..

[48] Ranson T., Vosshenrich C.A.J., Corcuff E., Richard O., Laloux V., Lehuen A., Di Santo J.P. (2003). IL-15 availability conditions homeostasis of peripheral natural killer T cells. Proc. Natl. Acad. Sci. USA.

[49] Takashima S., Martin M.L., Jansen S.A., Fu Y., Bos J., Chandra D., O’Connor M.H., Mertelsmann A.M., Vinci P., Kuttiyara J. (2019). T cell-derived interferon-gamma programs stem cell death in immune-mediated intestinal damage. Sci. Immunol..

[50] Nava P., Koch S., Laukoetter M.G., Lee W.Y., Kolegraff K., Capaldo C.T., Beeman N., Addis C., Gerner-Smidt K., Neumaier I. (2010). Interferon-gamma regulates intestinal epithelial homeostasis through converging beta-catenin signaling pathways. Immunity.

[51] Wingender G., Stepniak D., Krebs P., Lin L., McBride S., Wei B., Braun J., Mazmanian S.K., Kronenberg M. (2012). Intestinal microbes affect phenotypes and functions of invariant natural killer T cells in mice. Gastroenterol..

[52] Vahl J.C., Heger K., Knies N., Hein M.Y., Boon L., Yagita H., Polic B., Schmidt-Supprian M. (2013). NKT cell-TCR expression activates conventional T cells in vivo, but is largely dispensable for mature NKT cell biology. PLoS Biol..

[53] Olszak T., An D., Zeissig S., Vera M.P., Richter J., Franke A., Glickman J.N., Siebert R., Baron R.M., Kasper D.L., Blumberg R.S. (2012). Microbial Exposure During Early Life Has Persistent Effects on Natural Killer T Cell Function. Science.

[54] Ma C., McCallen J., McVey J.C., Trehan R., Bauer K., Zhang Q., Ruf B., Wang S., Lai C.W., Trinchieri G. (2023). CSF-1R+ Macrophages Control the Gut Microbiome-Enhanced Liver Invariant NKT Function through IL-18. J. Immunol..

[55] Ma C., Han M., Heinrich B., Fu Q., Zhang Q., Sandhu M., Agdashian D., Terabe M., Berzofsky J.A., Fako V. (2018). Gut microbiome-mediated bile acid metabolism regulates liver cancer via NKT cells. Science.

[56] Lin Q., Kuypers M., Baglaenko Y., Cao E., Hezaveh K., Despot T., de Amat Herbozo C., Cruz Tleugabulova M., Umaña J.M., McGaha T.L. (2024). The intestinal microbiota modulates the transcriptional landscape of iNKT cells at steady-state and following antigen exposure. Mucosal Immunol..

[57] An D., Oh S., Olszak T., Neves J., Avci F., Erturk-Hasdemir D., Lu X., Zeissig S., Blumberg R., Kasper D. (2014). Sphingolipids from a symbiotic microbe regulate homeostasis of host intestinal natural killer T cells. Cell.

[58] Carnaud C., Lee D., Donnars O., Park S.H., Beavis A., Koezuka Y., Bendelac A. (1999). Cutting edge: Cross-talk between cells of the innate immune system: NKT cells rapidly activate NK cells. J. Immunol..

[59] Wallace M.E. (1950). Locus of the gene 'fidget' in the house mouse. Nature.

[60] Exley M.A., Bigley N.J., Cheng O., Shaulov A., Tahir S.M., Carter Q.L., Garcia J., Wang C., Patten K., Stills H.F. (2003). Innate immune response to encephalomyocarditis virus infection mediated by CD1d. Immunology.

[61] Huang S., Hendriks W., Althage A., Hemmi S., Bluethmann H., Kamijo R., Vilcek J., Zinkernagel R.M., Aguet M. (1993). Immune response in mice that lack the interferon-gamma receptor. Science.

[62] Schindelin J., Arganda-Carreras I., Frise E., Kaynig V., Longair M., Pietzsch T., Preibisch S., Rueden C., Saalfeld S., Schmid B. (2012). Fiji: an open-source platform for biological-image analysis. Nat. Methods.

[63] Mootha V.K., Lindgren C.M., Eriksson K.F., Subramanian A., Sihag S., Lehar J., Puigserver P., Carlsson E., Ridderstråle M., Laurila E. (2003). PGC-1alpha-responsive genes involved in oxidative phosphorylation are coordinately downregulated in human diabetes. Nat. Genet..

[64] Subramanian A., Tamayo P., Mootha V.K., Mukherjee S., Ebert B.L., Gillette M.A., Paulovich A., Pomeroy S.L., Golub T.R., Lander E.S., Mesirov J.P. (2005). Gene set enrichment analysis: a knowledge-based approach for interpreting genome-wide expression profiles. Proc. Natl. Acad. Sci. USA.

[65] Bankhead P., Loughrey M.B., Fernández J.A., Dombrowski Y., McArt D.G., Dunne P.D., McQuaid S., Gray R.T., Murray L.J., Coleman H.G. (2017). QuPath: Open source software for digital pathology image analysis. Sci. Rep..

[66] Gracz A.D., Puthoff B.J., Magness S.T. (2012). Identification, isolation, and culture of intestinal epithelial stem cells from murine intestine. Methods Mol. Biol..

