# Supplementary material for: IFN-γ-dependent regulation of intestinal epithelial homeostasis by NKT cells
Source: Cell Rep. 2024 Nov 23;43(12):114948. doi: 10.1016/j.celrep.2024.114948 (PMC11876105; doi:10.1016/j.celrep.2024.114948)
Supplement: Document S1. Figures S1–S6 [file mmc1.pdf]

Cell Reports, Volume *43*

Supplemental information

IFN-γ-dependent regulation of intestinal epithelial homeostasis by NKT cells

Marta Lebrusant-Fernandez, Tom ap Rees, Rebeca Jimeno, Nikolaos Angelis, Joseph C. Ng, Franca Fraternali, Vivian S.W. Li, and Patricia Barral

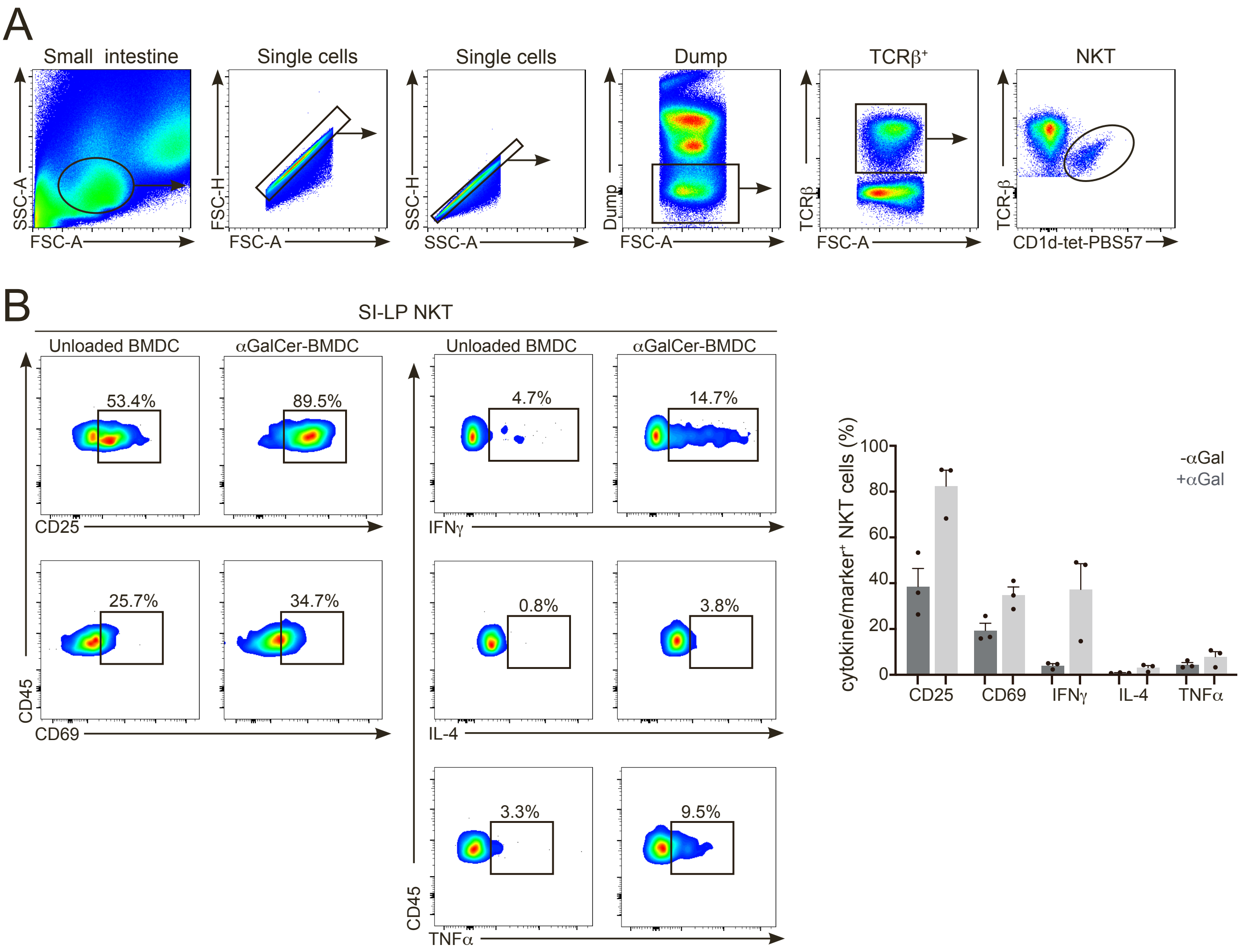

Supplementary Figure 1. Characterisation of SI-LP NKT cells. Related to Figure 1

(A) Gating strategy for SI-LP NKT cells.

(B) BMDCs were pulsed with 100ng/ml of αGalCer for 6h before overnight coculture with sort-purified NKT cells from SI-LP. NKT cell activation was measured by production of cytokines and expression of activation markers as indicated. Representative flow-cytometry plots (left) and quantifications (right) are shown. Data pooled from 2 independent experiments.

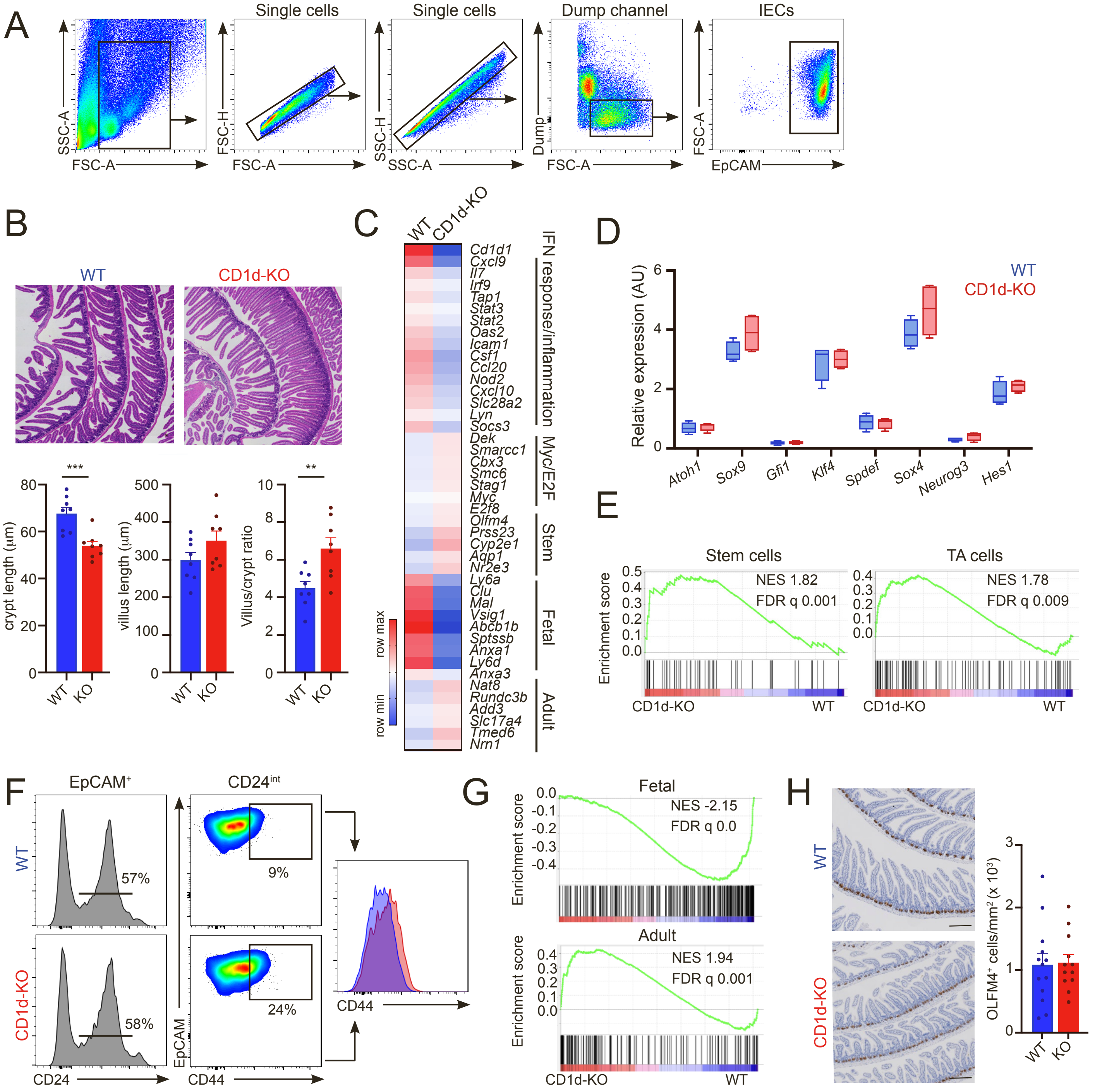

Supplementary Figure 2. Characterisation of WT and CD1d-KO IECs. Related to Figure 2

(A) Gating strategy for IECs.

(B) Haematoxylin and eosin (H&E) staining of proximal SI sections from CD1d-KO and WT mice (top) and quantification of crypt and villi length (bottom). Each dot is a mouse; bars represent mean +/- SEM. **p<0.01, ***p<0.001 unpaired t-test.

(C) Heatmap for fold-change of selected transcripts in CD1d-KO vs WT IECs.

(D) Relative gene expression of selected transcripts in IECs from WT (blue) and CD1d-KO (red) mice. Boxes show 25th to 75th percentiles with whiskers being max/min values.

(E) Enrichment plot for transcriptional signature for CD1d-KO (vs WT IECs) compared to signatures for stem[40] and transit amplifying (TA)[40] cells.

(F) Gating strategy and CD44 expression (in EpCAM+CD24int cells) for freshly isolated IECs.

(G) Enrichment plot for transcriptional signature of CD1d-KO (vs WT) IECs compared to signatures from foetal spheroids or adult organoids[44].

(H) Immunohistochemistry staining (left) and quantification (right) of OLFM4 positive cells in the SI of WT and CD1d-KO mice. Each dot is a quantified region with data pooled from 3 mice per group. Scale bar=250μm

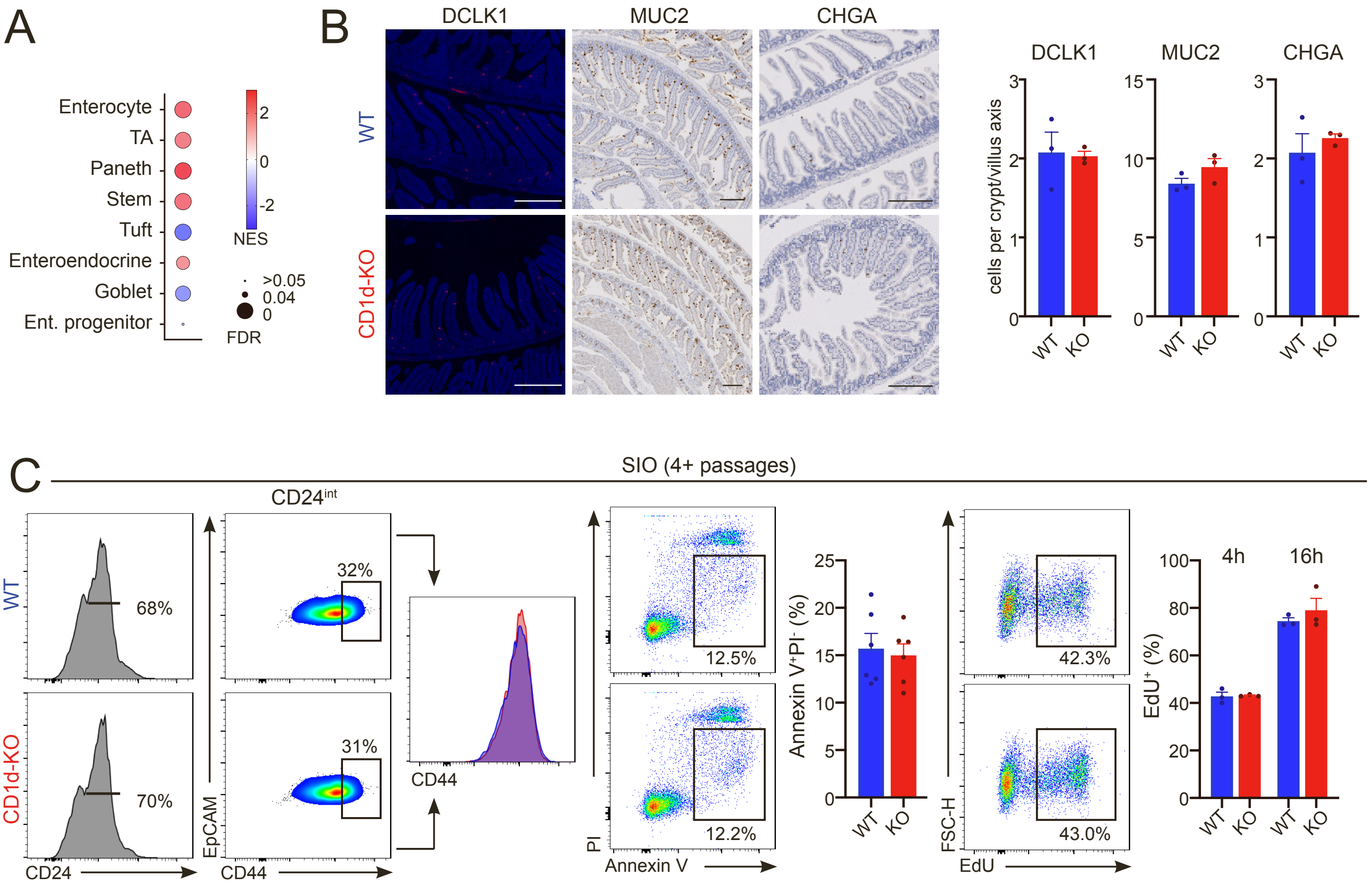

Supplementary Figure 3. Characterisation of WT and CD1d-KO IECs and established organoids. Related to Figure 2

(A) Enrichment plot for transcriptional signature of CD1d-KO (vs WT) IECs compared to signatures from various intestinal cell types[40,41].

(B) Staining and quantification of DCLK1 (Tuft cells), MUC2 (Goblet cells) and CHGA (enteroendocrine cells) in the SI of WT and CD1d-KO mice. n=3, each dot is a mouse. Scale bar=250μm

(C) WT and CD1d-KO established SIO (4+ passages) were analysed for CD44 expression (left), apoptotic cells (Annexin V, PI staining, middle) and proliferation (EdU incorporation, right). Organoids used for experiments were generated from 3 mice and data collected in at least 2 independent experiments. Bars represent mean +/- SEM.

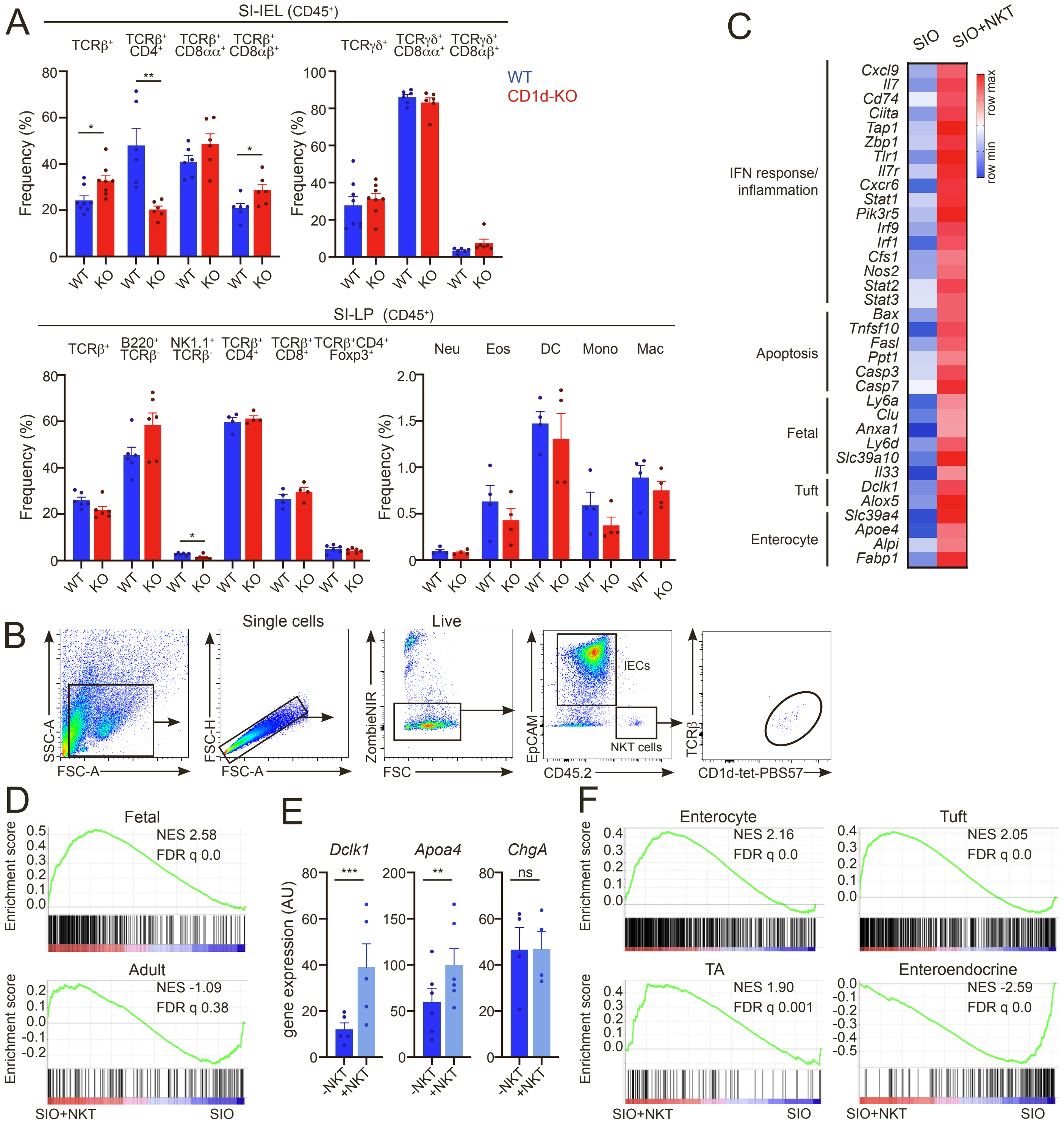

Supplementary Figure 4. Characterisation of intestinal immune cells and SIO after NKT cell co-culture. Related to Figure 3

(A) Frequency of lymphoid and myeloid populations determined by flow cytometry and shown as percentage of $CD45^{+}$ cells, within the intraepithelial lymphocyte fraction (top) and lamina propria (bottom) in the SI from WT and CD1d-KO mice. Lymphoid populations include T cells ($TCR\beta^{+}$), B cells ($B220^{+}TCR\beta^{-}$), NK cells ($NK1.1^{+}TCR\beta^{-}$), Tregs ($TCR\beta^{+}CD4^{+}Foxp3^{+}$). Myeloid populations include neutrophils (Neu, $Ly6G^{+}CD11b^{+}$), eosinophils (Eos, Siglec-$F^{+}CD11b^{+}Ly6G^{-}$), dencritic cells (DC, $CD11c^{+}MHCII^{+}Ly6G^{-}CD64^{-}F4/80^{-}$), monocytes (Mono, $Ly6C^{+}CD11b^{+}$) and macrophages (Mac, $CD64^{+}CD11b^{+}$Siglec-$F^{-}Ly6G^{-}$). Each dot is a mouse. Bars represent mean+/-SEM. *p<0.05, **p<0.01, unpaired t-test

(B) Gating strategy for IECs and NKT cells after SIO-NKT cell co-culture.

(C) Heatmap for fold-change of selected transcripts for SIO cultured alone or in the presence of NKT cells (SIO+NKT).

(D) Enrichment plot for transcriptional signature of SIO+NKT (vs SIO) compared to signatures from foetal spheroids or adult organoids[44].

(E) SIO were cultured in the presence (+NKT) or absence (-NKT) of SI-LP NKT cells as indicated. Gene expression was measured by qPCR. Organoids used for experiments were generated from 3 mice and data collected in at least 2 independent experiments. Bars represent mean+/-SEM. **p<0.01, ***p<0.001, paired t-test

(F) Enrichment plot for transcriptional signature of SIO+NKT (vs SIO) compared to signatures from the indicated cell types[40,41].

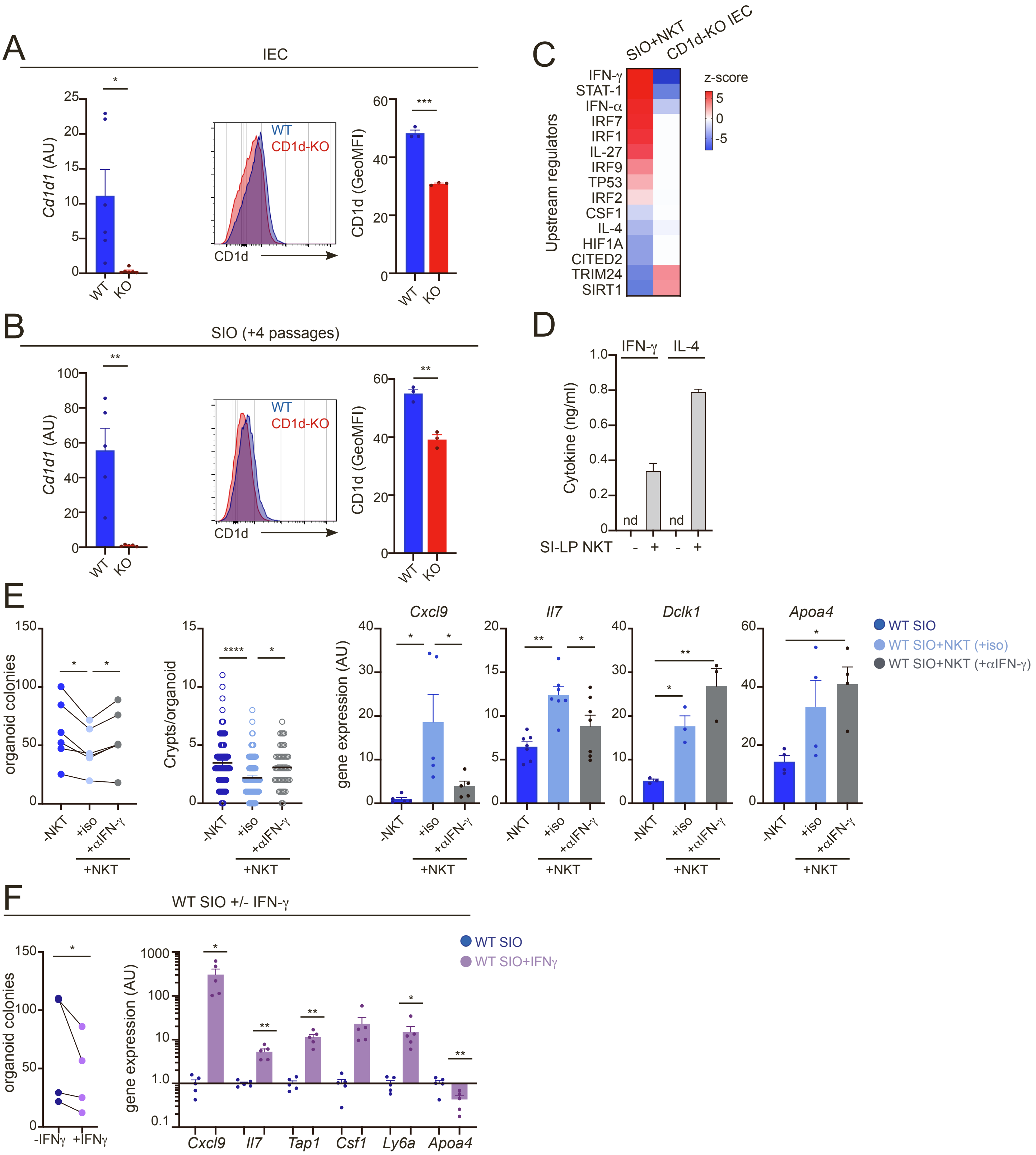

Supplementary Figure 5. NKT cell-IEC crosstalk. Related to Figure 4

(A-B) Expression of CD1d determined by qPCR (left) or flow-cytometry (right, quantified as geometric mean fluorescence intensity) in freshly isolated IECs (A) or SIO (B). n=3-6 each dot is a mouse (A) or organoids generated from one mouse (B). Bars represent mean+/-SEM. *p<0.05, **p<0.01, ***p<0.001; unpaired t-test

(C) Ingenuity pathway analysis of cytokine and transcription factors upstream regulators predicted to be driving expression signatures in SIO+NKT cells and CD1d-KO IECs.

(D) Cytokine secretion by NKT cells sort-purified from the SI-LP. Cytokines in the culture supernatant were quantified by cytometric bead array. Representative data from 3 independent experiments is shown

(E) WT SIO were cultured in the presence (+NKT) or absence (-NKT) of NKT cells and αIFN-γ (or isotype control) as indicated. Quantification of SIO (left), crypts per organoid (middle), and gene expression (rigth) are shown. Organoids used for experiments were generated from 3 mice and data collected in 2-3 independent experiments. Bars represent mean+/-SEM. *p<0.05, **p<0.01, ****p<0.0001; one-way ANOVA with Tukey's multiple comparisons.

(F) WT SIO were cultured in the presence of IFN-γ. Number of SIO recovered after culture with IFN-γ (left) and expression of the indicated genes measured by qPCR (right). Bars represent mean+/-SEM. *p<0.05, **p<0.01; paired (left) or one sample (right) t-test.

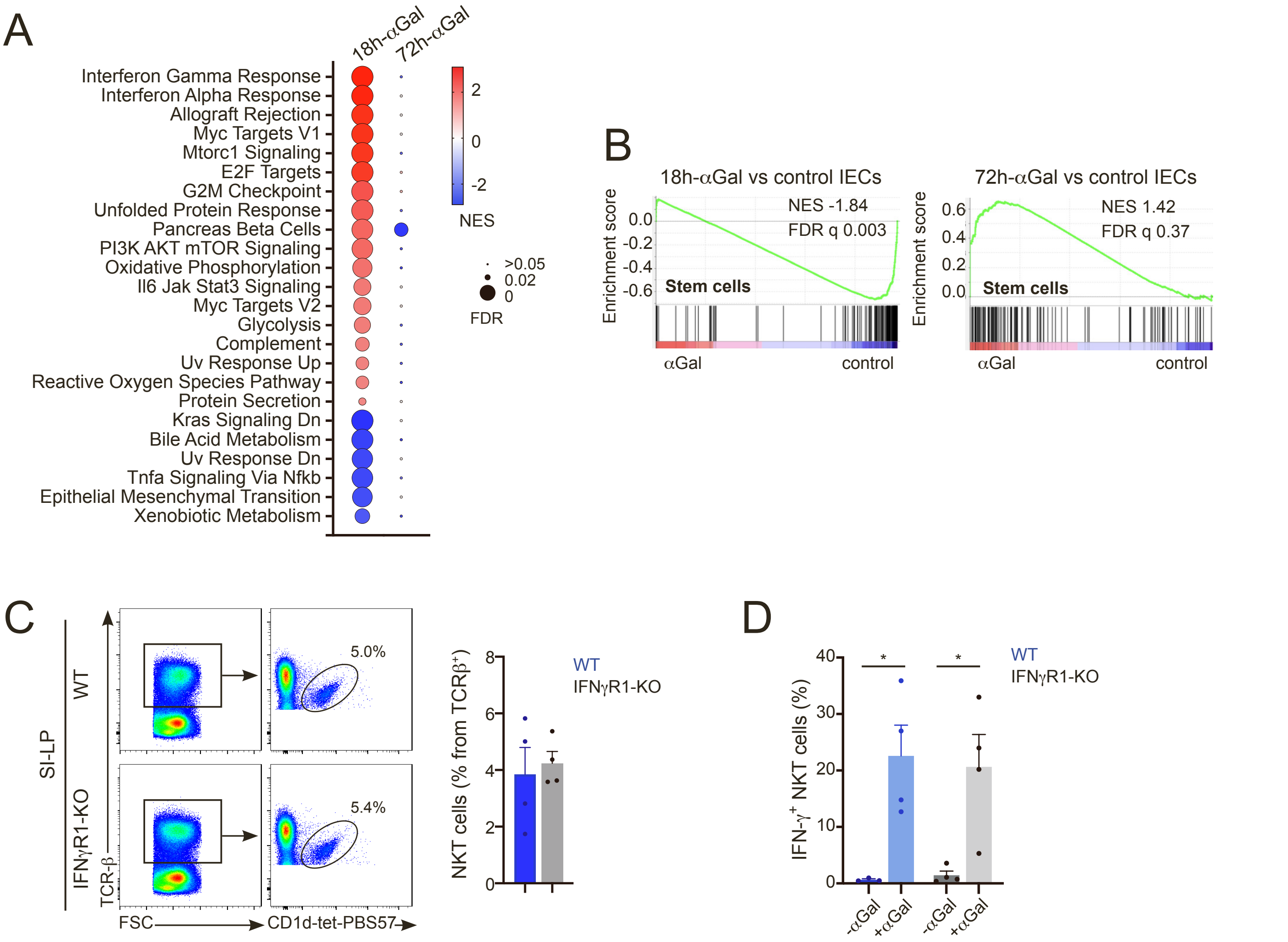

Supplementary Figure 6. NKT cell activation *in vivo* regulates IECs. Related to Figure 5

(A) Results of GSEA Hallmark analysis showing enriched gene sets for 18h-αGal or 72h-αGal vs control IECs.

(B) Enrichment plot for transcriptional signature for 18h-αGal or 72h-αGal (vs control IECs) compared to signatures from stem cells[41].

(C) Flow cytometry plots (left) and quantification (as frequency of TCRβ$^+$ cells, right) for NKT cells in the small intestinal lamina propria of WT and IFNγR1-KO mice as indicated.

(D) Mice were injected with αGalCer (+αGal) or PBS (-αGal) and SI-LP NKT cells were analysed 5h later. Quantification of IFN-γ secreting NKT cells in WT or IFNγR1-KO mice is shown.

Bars represent mean +/- SEM. Each dot is a mouse, and data are pooled from 3 independent experiments. *p<0.05 unpaired t-test
